# Salt tolerance characterization and genome-wide association study of *Gossypium barbadense* accessions reveal salinity-adaptive variations

**DOI:** 10.3389/fpls.2025.1654742

**Published:** 2025-09-11

**Authors:** Huazu Li, Shuhui Wang, Zhengning Zheng, Yue Sun, Yifei Han, Mengyu Xing, Tianxu Zhang, Wenlong Mo, Binbin Cai, Jinghan Yin, Jiajie Qian, Uzair Muhammad, Wei Li, Daojun Yuan, Jinhong Chen, Shuijin Zhu, Tianlun Zhao

**Affiliations:** ^1^ College of Agriculture and Biotechnology, Zhejiang University, Hangzhou, China; ^2^ Hainan Institute, Zhejiang University, Sanya, China; ^3^ Zhejiang Agricultural Technical Extension Center, Hangzhou, China; ^4^ Institute of Crop and Nuclear Technology Utilization, Zhejiang Academy of Agricultural Sciences, Hangzhou, China; ^5^ Chinese Academy of Agricultural Sciences, State Key Laboratory of Cotton Bio-breeding and Integrated Utilization, Anyang, China; ^6^ Zhengzhou Research Base, State Key Laboratory of Cotton Bio-breeding and Integrated Utilization, School of Agricultural Sciences, Zhengzhou University, Zhengzhou, China; ^7^ Engineering Research Centre of Cotton of Ministry of Education, College of Agronomy, Xinjiang Agricultural University, Urumqi, Xinjiang, China; ^8^ National Key Laboratory of Crop Genetic Improvement, Huazhong Agricultural University, Wuhan, Hubei, China

**Keywords:** *Gossypium barbadense*, seedling stage, salt tolerance index, salt tolerance, genomewide association analysis

## Abstract

**Introduction:**

As a globally important cash crop, *Gossypium barbadense* has the high-quality fiber for textile industry. However, it experiences substantial growth inhibition and yield decline under salt stress, rendering the elucidation of its salt tolerance mechanisms imperative for breeding initiatives.

**Methods:**

We performed population structure analysis on 240 global *G. barbadense* accessions, phenotyping under salt stress at seedling-stage, genome-wide association study (GWAS), virus-induced gene silencing (VIGS) of *Gbar_D02G014670* (*GbXTH27*), and its functional verification.

**Results:**

Population structure analysis on 240 globally distributed *G. barbadense* accessions resolved four distinct subpopulations. Seedling-stage salt stress screening identified 23 highly salt-tolerant genotypes exhibiting divergent phenotypic responses. GWAS identified multiple significant single nucleotide polymorphism (SNP) loci associated with salt tolerance, with the most prominent signal localized to chromosome D02. VIGS of *GbXTH27* exacerbated salt-induced wilting phenotypes and significantly decreased antioxidant enzyme activities.

**Discussion:**

This research provides valuable molecular markers and theoretical foundations for genetic improvement and breeding of salt-tolerant *G. barbadense* cultivars, while also offering insights into salt stress response mechanisms applicable to other crops.

## 1 Introduction

Cotton (*Gossypium* spp.) is a globally significant cash crop, particularly in the case of *G. barbadense*, has garnered substantial attention from the textile industry and breeders due to its superior fiber quality and disease resistance. As the exclusive cotton cultivation region in the China, Xinjiang benefits from unique geographical and climatic conditions (Zhao et al., 2024). However, it faces severe challenges from soil salinization, which critically impairs cotton growth, yield, and fiber quality (Sharif et al., 2019; Su et al., 2020; Zhu et al., 2020). To enhance or stabilize yield and fiber quality, breeding salt-tolerant *G. barbadense* cultivars has emerged as a pivotal objective in cotton improvement programs.

Salt tolerance, a polygenic trait essential for plant adaptation to saline environments, involves coordinated regulation of multiple quantitative characteristics including plant height (Wang et al., 2012; Long et al., 2013), root length (Li et al., 2021; Lin et al., 2004), biomass (Seemann and Critchley, 1985), organic osmolyte accumulation (Duan et al., 2023), and ion homeostasis (Lin et al., 2004). To systematically evaluate these traits, the membership function value (MFV) methodology has been established as a quantitative framework integrating growth parameters, leaf injury indices, and ion concentrations under salt stress. For instance, MFV-based screening of 549 *Brassica napus* inbred lines during germination stages identified salt-tolerant genotypes using germination rate, root/shoot length, and fresh weight (Wu et al., 2019). Similarly, 300 sweet sorghums (*Sorghum bicolor (L.) Moench*.) accessions were classified for salt tolerance at germination using MFV indices derived from five traits including germination energy, germination rate, germination index, germination vigour index and root fresh weight (Ding et al., 2018). In sunflower (*Helianthus annuus* L.), MFV combined with principal component analysis (PCA) generated a Composite Stress Assessment Index (CSAI) to evaluate multi-stress responses (Ma et al., 2016).

Genome-wide association studies (GWAS) have emerged as a powerful tool for dissecting the genetic architecture of agronomic traits in crops, facilitating the identification of key loci governing yield and quality. GWAS has been effectively applied in major crops, including *Oryza sativa* (Zhao et al., 2011; Huang et al., 2012), *Glycine max* (Zhang et al., 2015; Zhao et al., 2019), *Brassica napus* (Schiessl et al., 2015; Lu et al., 2017), *G. hirsutum* (Fang et al., 2017; Huang et al., 2017). Based on these successes, GWAS has been increasingly applied to unravel the complex mechanisms underlying salt stress tolerance, with significant progress achieved in major crops. In *Oryza sativa* (Li et al., 2020; Wei et al., 2024), *Triticum aestivum* (Hu et al., 2021; Quamruzzaman et al., 2022b), *Zea mays* (Li et al., 2021, 2022), *Glycine max* (Do et al., 2019; Jin et al., 2021), and *Brassica napus* (Zhang et al., 2022, 2023), numerous salt-stress QTLs and candidate genes have been identified through GWAS analyses. The recent advancements in high-throughput sequencing technologies and the availability of refined genome assemblies for *Gossypium* species (Hu et al., 2019; Wang et al., 2019) have further expanded the application of GWAS in cotton, particularly for elucidating the genetic basis of salt tolerance mechanisms. A total of 42 salt-tolerance-associated SNPs were detected in 149 *G. hirsutum* accessions using the Illumina Cotton SNP70K array, and genes involved in intracellular transport, sucrose synthesis, and auxin response were revealed (Zheng et al., 2021). Eight significant SNPs linked to three salt-stress traits were identified through Cotton SNP80K chip analysis of 288 *G. hirsutum* accessions (Cai et al., 2017). Genotyping-by-sequencing (GBS) based GWAS of 217 *G. hirsutum* varieties identified *GH_A13G0171* as a negative regulator of salt response (Xu et al., 2021). Resequencing of 215 *G. arboreum* accessions revealed nine SNP-rich regions and 40 candidate genes (Dilnur et al., 2019). Integrating RNA-seq and GWAS of 214 Chinese *G. arboreum* accessions, Transcriptome-wide association study (TWAS) in *G. hirsutum* seedlings pinpointed 19 salt-responsive genes (Han et al., 2022).

Despite these advancements, research on salt tolerance mechanisms in *G. barbadense* remains limited compared to *G. hirsutum* (Xu et al., 2023; Zhang et al., 2024). To address this gap, we constructed a high-density genetic variation map using 240 globally collected *G. barbadense* accessions. Through two-year seedling-stage salt stress trials and phenotypic characterization, combined with GWAS, we identified key loci associated with salt tolerance and functionally validated candidate genes. This work provides molecular markers and target genes for genetic enhancement of salt tolerance in *G. barbadense*, while providing methodological references for dissecting mechanism of stress tolerance in other crops.

## 2 Materials and methods

### 2.1 Experimental materials

A total of 240 *G. barbadense* accessions from diverse countries and regions were collected for seedling-stage salt tolerance evaluation. These included 220 mainstream cultivars from Xinjiang, China, three wild *G. barbadense* accessions collected from Yunnan and Hainan, China, six Pima cotton germplasm lines from the United States, six cultivated materials from Egypt, and five *G. barbadense* varieties from Central Asia.

### 2.2 Phenotypic evaluation and analysis

Sulfuric acid-delinted seeds were sown in 10 × 5 seedling trays. After 3 days of germination, seedlings were transferred to a hydroponic system containing 1/2-strength Murashige and Skoog (MS) nutrient solution (pH 5.8). The nutrient solution was replaced every 3 days, and continuous aeration was maintained using an air pump. Plants were grown under controlled environmental conditions in the greenhouse at Zhejiang University Agricultural Experiment Station. NaCl treatment (200 mmol/L) was initiated at the two true leaves and one apical bud stage, while control groups remained untreated, both the control groups and the salt-stress groups were cultured synchronously in the hydroponic system. After 7 days of salt stress, senesced cotyledons were removed. Plant height (cotyledonary node to apical meristem) and shoot fresh weight were measured. Roots and shoots were then oven-dried at 105 °C for 60 min followed by 80 °C to constant weight for dry weight determination. Three biological replicates per treatment were maintained to ensure experimental reliability. All measured parameters were converted to the Salt Tolerance Index (STI), which was calculated using Equation 1:


(1) $$\begin{array}{c} STI=Measurement\;under\;salt\;stress/\\ Measurement\;under\;control\;conditions\; \end{array}$$

The traits included relative plant height (RPH), relative shoot fresh weight (RSFW), relative shoot dry weight (RSDW), and relative root dry weight (RRDW). To minimize environmental variance across years and emphasize genetic effects, best linear unbiased estimates (BLUEs) for four traits (2022–2023 data) were calculated using the lme4 R package:


(2) $$\begin{array}{c} BLUE=lmer(STI\\ ∼Sample+(1|Rep)+(1|Year:Rep)+(1|Year)) \end{array}$$

In Equation 2, *STI* serves as the dependent variable, with Sample (genotype) designated as the fixed-effect independent variable. The term *1|Rep* denotes experimental replicates modeled as random effects, *1|Year* represents year-specific random effects, and *1|Year: Rep* specifies the nested random effects of replicates within years.

BLUE values were analyzed for descriptive statistics and ANOVA using SPSS v26. Phenotypic frequency distributions and correlations were visualized with the Hmisc R package. Graphs were generated using GraphPad Prism.

### 2.3 Salt tolerance assessment

A membership function method was applied to comprehensively evaluate seedling-stage salt tolerance across traits.


(3) $$F_{i}=\sum_{j=1}^{4}[E_{ij}\times STI_{j}]$$

(4) $$\mu (F_{i})=(F_{i}-F_{imin})/(F_{imax}-F_{imin})\;$$

(5) $$W_{i}=P_{i}/\sum_{i=1}^{n}P_{i}$$

(6) $$D=\sum_{i=1}^{n}[\mu (F_{i})\times W_{i}]$$

In Equation 3, $F_{i}$ denotes the comprehensive index factor score of genotype *i*. $E_{ij}$ represents the eigenvector corresponding to the *j*-th individual indicator in the *i*-th principal component. $STI_{j}$ indicates the salt tolerance index of the *j*-th individual indicator for genotype *i*. In Equation 4, $\mu (F_{i})$ refers to the membership value of the comprehensive index for genotype *i*, where $F_{imax}$ and $F_{imin}$ represent the maximum and minimum values of the comprehensive index, respectively. Equation 5 defines $W_{i}$ as the weight (relative importance) of the *i*-th comprehensive index among all indices, calculated from its contribution rate ( $P_{i}$ ). In Equation 6, *D* quantifies the integrated salt tolerance coefficient.

Correlation analysis was performed using SPSS. Hierarchical clustering analysis was conducted in R with the hclust function, employing Euclidean distance and Ward’s minimum variance method for cluster aggregation.

### 2.4 Measurement of superoxide dismutase activities, malondialdehyde and proline content

Oxidative stress markers, including superoxide dismutase (SOD) activities, malondialdehyde (MDA) and proline (Pro) content were measurement using established protocols outlined (Qian et al., 2024). All assays utilized a 0.1 g fresh sample of leaves.

### 2.5 Variant calling and population genetics analysis

Resequencing data PRJNA728217 (Yu et al., 2021) for 240 *G. barbadense* accessions was downloaded from the NCBI SRA database using SRA Toolkit. Raw Illumina paired-end reads were quality-filtered using Fastp (Chen et al., 2018), with parameters “-c -n 15 -u 50 -q 15” to retain high quality sequences (Shao et al., 2022). Clean reads were aligned to the *G. barbadense* (AD2) ‘3-79’ reference genome HAU (Wang et al., 2019) using BWA (Li and Durbin, 2009), following index construction with the same tool. Alignment files were converted to binary BAM format using SAMtools (Li et al., 2009), sorted with sambamba (Tarasov et al., 2015), and PCR duplicates were removed.

SNPs and InDels were identified using the HaplotypeCaller module in GATK (Mckenna et al., 2010). GVCF files were merged with CombineGVCFs and converted to VCF format. Variants were filtered using VariantFiltration module in GATK (Mckenna et al., 2010), followed by additional filtering (maf > 0.05, max-missing > 0.8) to obtain GWAS-compatible SNPs (Yu et al., 2021). Functional annotation was performed using ANNOVAR. SNP/InDel densities were calculated with VCFtools (Danecek et al., 2011), and chromosomal distributions were visualized using RColorBrewer and stringr R packages.

A genetic distance matrix generated by VCF2Dis was used to construct a Neighbor-Joining (NJ) phylogenetic tree via the FastME online platform (http://www.atgc-montpellier.fr/fastme). Population structure was inferred using Admixture (Alexander et al., 2009), and PCA was conducted with GCTA (Yang et al., 2011) to resolve substructure and mitigate false positives in association studies. Genome-wide linkage disequilibrium (LD) decay was assessed with PopLDdecay (Zhang et al., 2019a).

To quantify genetic divergence and variation among subpopulations, pairwise population differentiation index (*F*_ST_) and nucleotide diversity (π) were calculated genome-wide using VCFtools (Danecek et al., 2011) with 100-kb sliding windows and 20-kb steps.

### 2.6 Genome-wide association study

Three types of salt stress phenotypic data for the seedling stage of *G. barbadense* were generated based on the calculated BLUE values and the phenotypes observed in 2022 and 2023. The genome-wide efficient mixed-model association (GEMMA) software (Zhou and Stephens, 2012), was used to correct for population stratification by incorporating both PCA and kinship matrices. Manhattan plots were generated using the R package CMplot to represent the distribution of SNPs and their corresponding P-values, while quantile-quantile (QQ) plots were constructed to evaluate the model’s performance. SNP filtering was performed using Plink software based on linkage disequilibrium criteria (window size = 50, step size = 50, r² ≥ 0.2), resulting in a total of 213,990 effective SNPs. A stringent threshold of *p* < 4.67 × 10^-6^ was set to identify significant association loci (Yu et al., 2021). However, due to the risk of overly stringent thresholds excluding true trait-associated genetic loci with *p*-values that do not meet the strict cutoff, SNPs with *p* < 1.0 × 10^-5^ identified in at least two environments or phenotypes were also retained to capture more candidate genes (Zhao et al., 2022).

### 2.7 RNA-seq analysis

RNA-seq data were downloaded from NCBI under project numbers PRJNA490626 (Hu et al., 2019) (salt stress treatments at 1 h, 3 h, 6 h, 12 h, and 24 h) and PRJNA601953 (salt stress treatment at 14 days) (Dong et al., 2022). Additionally, the *G. barbadense* genome (version 379_HAU) was obtained from COTTONGENE (https://www.cottongen.org/species/Gossypium_barbadense/nbi-AD2_genome_v1.0) and the genome index was built using ​​HISAT2 (Kim et al., 2019). Quality control and filtering were performed using ​​Fastp​​ (Chen et al., 2018) with the following criteria (Song et al., 2023): paired reads were removed if any read met the following criteria: ambiguous “N” bases exceeded 10% of the read length; >50% of bases had low quality (Q ≤ 5); or adapter sequences were detected. Reads were aligned to the reference genome using SAMtools (Li et al., 2009), and gene expression levels were quantified as FPKM values using StringTie (Pertea et al., 2015).

### 2.8 Gene expression analysis

Two contrasting materials, the highly salt-tolerant line H160 and the salt-sensitive line H20, were subjected to salt stress (200 mmol/L NaCl) at 0 h, 24 h, and 48 h, with three biological replicates per time point. Total RNA was extracted from leaf samples using an RNA extraction kit (TIANGEN), reverse-transcribed into cDNA using a reverse transcription kit (TOROIVD), and subjected to qRT-PCR analysis using enzymes from TOROGreen^®^ qPCR Master Mix (TOROVID). The qPCR reactions were performed in a 20 μL reaction system containing 2 μL of cDNA, 10 μL of qPCR master mix, 4 μL of forward primer, and 4 μL of reverse primer, with each primer at a final concentration of 1–2 micromolars. The housekeeping gene *UBQ7* (Ubiquitin extension protein 7) was employed as an internal control gene (Wang et al., 2013). Each template was analyzed in triplicate technical replicates. The qPCR protocol was conducted using the LightCycler 96 real-time PCR system (Roche) with the following cycling conditions: initial denaturation at 96°C for 3 minutes, followed by 40 cycles of denaturation at 95°C for 10 seconds and annealing/extension at 60°C for 30 seconds. Primers used for quantitative analysis are listed in **Supplementary Table S1**.

### 2.9 VIGS experiment

Virus-induced gene silencing (VIGS) was performed using the tobacco rattle virus (TRV)-based pTRV1/2 vector system.​​ Target gene fragments were amplified from *G. barbadense* cDNA and cloned into the pTRV2 vector via homologous recombination using *EcoR*I and *Kpn*I restriction sites. The constructed vectors were transformed into Escherichia coli DH5α cells and validated by plasmid sequencing. Verified vectors were introduced into Agrobacterium tumefaciens strain GV3101 via heat-shock transformation. ​​Agrobacterial cultures​​ harboring the vectors were grown in liquid medium supplemented with rifampicin (50 μg/mL) and kanamycin (50 μg/mL) at 28°C with 200 rpm agitation for 12 h. Bacterial cells were harvested, resuspended in infiltration buffer (10 mM MgCl2, 10 mM MES, 200 μM acetosyringone), and adjusted to OD600 = 1.0. After 2–3 h of dark incubation at 28°C, Agrobacterium suspensions carrying pTRV1 and pTRV2 (negative control), pTRV1 and pTRV2: *CLA1* (positive control), or pTRV1 and pTRV2: *GbXTH27* were mixed at 1:1 ratio. The mixtures were infiltrated into cotyledons of 7-day old *G. barbadense* 3–79 seedlings using sterile syringes. ​​Post-infiltration​​, plants were maintained in darkness for 24 h, then transferred to a growth chamber at 25°C under 16 h light/8 h dark cycles. The empty pTRV2 vector served as a negative control, while pTRV2: *CLA1* (essential for chloroplast development) was used as a positive control, inducing characteristic leaf whitening within two weeks due to chloroplast defects (Mandel et al., 1996). Leaves from pTRV2 and pTRV2: *GbXTH27* infiltrated plants were collected at two weeks post-infiltration for RNA extraction and qPCR validation of *GbXTH27* silencing efficiency. ​​All primers used for VIGS vector construction are listed in **Supplementary Table S1**.

### 2.10 DAB staining

The DAB solution was formulated by dissolving DAB powder in distilled water to reach a concentration of 1 mg/mL, with its pH adjusted to 3.8. For the staining procedure, leaves were immersed in the prepared DAB solution and incubated at 28°C in the dark for 12 hours. Following this, the leaves underwent a 10-minute boiling treatment in 95% ethanol to remove chlorophyll.

### 2.11 Determination of elements

Sodium (Na) and potassium (K) contents were measured via inductively coupled plasma atomic emission spectroscopy (ICP-AES) (Zhang et al., 2019b). Roots, stems, and leaves of cotton seedlings were collected, dried, and ground to pass through a 40-mesh sieve. The homogenized samples were thoroughly mixed prior to analysis to ensure representativeness.

## 3 Results

### 3.1 Construction of the *G. barbadense* variation map

This study employed a population of 240 *G. barbadense* accessions to investigate genetic variation and construct a high-density variation map. A total of 2,983,855 high-quality SNPs were identified, which were unevenly distributed across chromosomes (**Supplementary Figure S1**, **Supplementary Table S2**). The At and Dt subgenomes contained 1,947,267 and 1,036,588 SNPs, respectively, with the At subgenome harboring approximately 1.88 times more SNPs than the Dt subgenome, which was consistent with the At subgenome being roughly twice the size of the Dt subgenome (Hu et al., 2019). The average SNP density across the genome was 1.40 SNPs/kb, with densities of 1.45 SNPs/kb and 1.32 SNPs/kb in the At and Dt subgenomes, respectively. Chromosome A07 showed the highest SNP density (4.35 SNPs/kb), followed by D10 (3.48 SNPs/kb). Conversely, A03 exhibited the lowest density (0.62 SNPs/kb), with D12 marginally higher (0.64 SNPs/kb). Annotation of SNPs using ANNOVAR revealed 29,495 non-synonymous SNPs, 16,606 synonymous SNPs, 136,073 upstream/downstream SNPs, 741 stop-gain SNPs, 112 stop-loss SNPs, and 356 splicing SNPs (**Supplementary Table S3**). Linkage disequilibrium (LD) decay was estimated using the r² coefficient between SNPs. LD decay distances at which r² dropped to half-maximum (0.5) were approximately 3,000 kb for the whole genome, with 5,200 kb for the At subgenome and 1,300 kb for the Dt subgenome (**Supplementary Figure S2**). The slower LD decay in the At subgenome compared to the Dt subgenome may reflect differential selection pressures during domestication.

### 3.2 Population structure analysis of *G. barbadense* population

**Figure 1:**
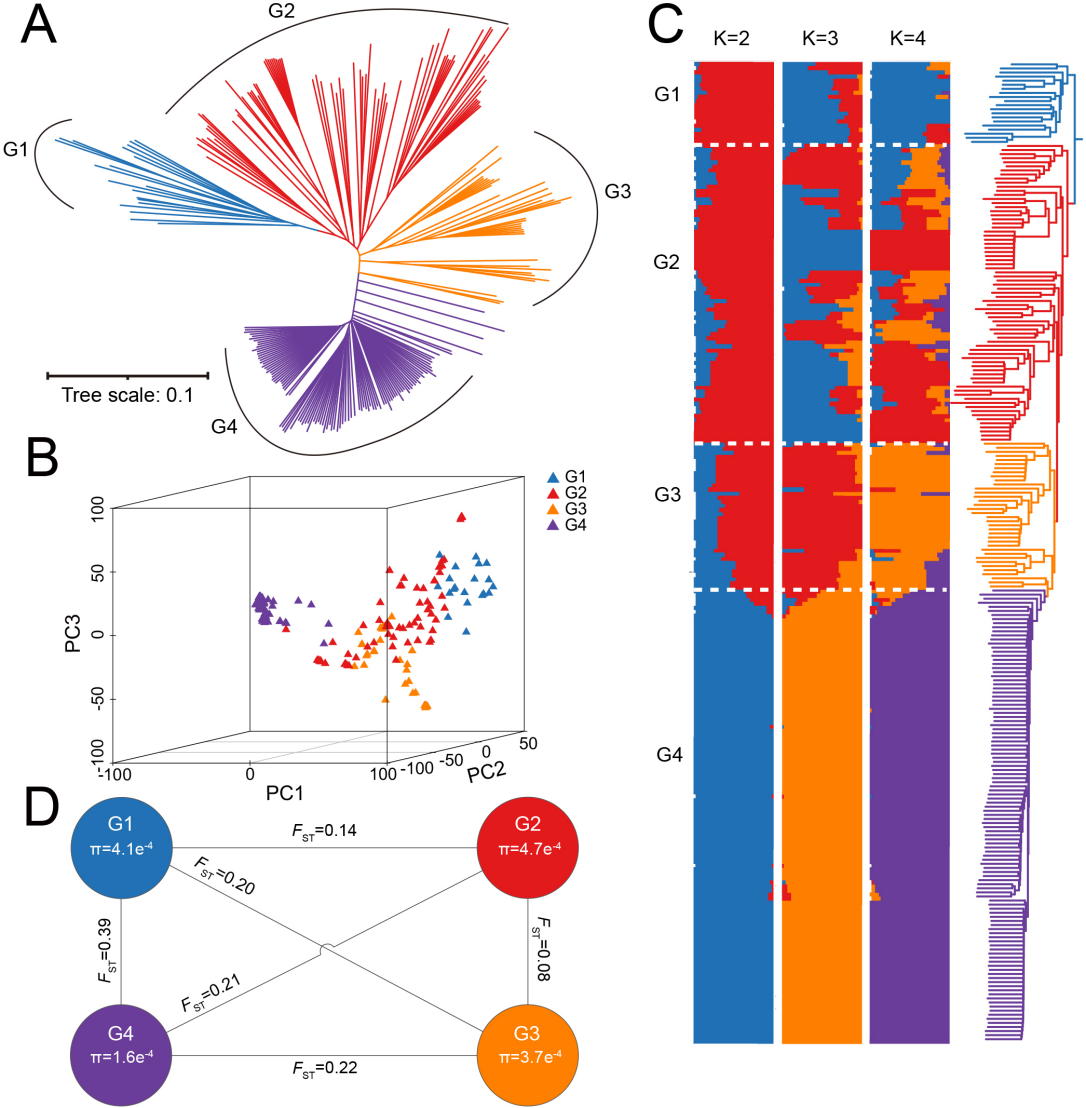
Population genetic structure of 240 *G. barbadense*. **(A)** Neighbor-joining phylogenetic tree of 240 *G. barbadense* accessions clustered into four subgroups (G1-G4). Branch colors correspond to genetic subgroups. **(B)** PCA of 240 accessions visualized in three dimensions. Points are colored by genetic subgroup (G1-G4). **(C)** Admixture ancestry proportions for K = 2-4. Vertical bars represent individual accessions, partitioned into genetic subgroups (G1-G4) at optimal K = 4. **(D)** *F*_ST_ and π across subgroups G1-G4.

To investigate the origin, genetic diversity, and differentiation among subpopulations in the *G. barbadense* population, phylogenetic tree construction, population structure analysis, and PCA were performed. Population structure is a major factor influencing GWAS results and can lead to false positives (Price et al., 2006). Therefore, PCA and kinship (**Supplementary Figure S3**) matrices were incorporated to correct for population stratification and reduce spurious associations. A neighbor-joining (NJ) phylogenetic tree divided the population into four subgroups, including G1 (20 accessions), G2 (73 accessions), G3 (36 accessions), and G4 (111 accessions) (**Figure 1A**, **Supplementary Table S4**). All G1 accessions originated from regions outside Xinjiang, China. Cross-validation (CV) results showed the lowest error at K = 8, with CV errors stabilizing from K = 4 onward (**Supplementary Figure S4**). PCA results were consistent with the phylogenetic tree, dividing the 240 accessions into four groups (**Figure 1B**). Admixture analysis at K = 4 confirmed these findings, showing that accessions from regions outside Xinjiang clustered together. Notably, G4 was distinct even at K = 2, with no admixture from other groups. This indicates low genetic diversity and limited hybridization, likely due to independent artificial selection during breeding (**Figure 1C**). The *F*_ST_ and π ratios were calculated for each subgroup using SNP data to assess the differences among groups. The *F*_ST_ value between G1 and G2 was the highest (0.39), while the *F*_ST_ value between G2 and G3 was the lowest (0.08), suggesting frequent genetic exchange between G2 and G3 during breeding process. G2 exhibited the highest nucleotide diversity (π = 4.7×10^-4^), indicating greater genetic resources, whereas G4 had the lowest π (1.6×10^-4^) (**Figure 1D**). These results highlight significant differences among the four subgroups, with G4 showing the lowest genetic diversity and experiencing the strongest selection pressure, which provides valuable insights into the breeding history of *G. barbadense*.

### 3.3 Salt tolerance evaluation of *G. barbadense* population

Data of four traits were collected from 239 *G. barbadense* accessions under 200 mmol/L NaCl salt stress in both 2022 and 2023. The traits included relative plant height (RPH), relative shoot fresh weight (RSFW), relative shoot dry weight (RSDW), and relative root dry weight (RRDW). To minimize the influence of environmental and batch effects, the best linear unbiased estimates (BLUEs) for each trait were calculated (**Table 1**). BLUE-adjusted trait values showed differential sensitivity to salinity: RPH (0.49-0.79, mean ± SD = 0.62 ± 0.05), RSFW (0.27-0.79, 0.49 ± 0.09), RSDW (0.35-0.88, 0.58 ± 0.10), RRDW (0.35-0.85, 0.59 ± 0.08). All four metrics were less than 1, indicating that the 200 mmol/L NaCl treatment reduced plant height, shoot fresh weight, shoot dry weight, and root dry weight during the seedling stage of *G. barbadense*. Among the traits, the coefficient of variation (CV) of RPH was the smallest (7.99%) and RSFW was the largest (18.42%).

**Table 1:** Salt-stressed phenotypic data for 239 *G. barbadense* accessions.

Trait	Max	Min	Mean	SD	CV(%)
Relative plant height	0.79	0.49	0.62	0.05	7.99
Relative shoot fresh weight	0.79	0.27	0.49	0.09	18.42
Relative shoot dry weight	0.88	0.35	0.58	0.10	16.90
Relative root dry weight	0.85	0.35	0.59	0.08	13.66

**Figure 2:**
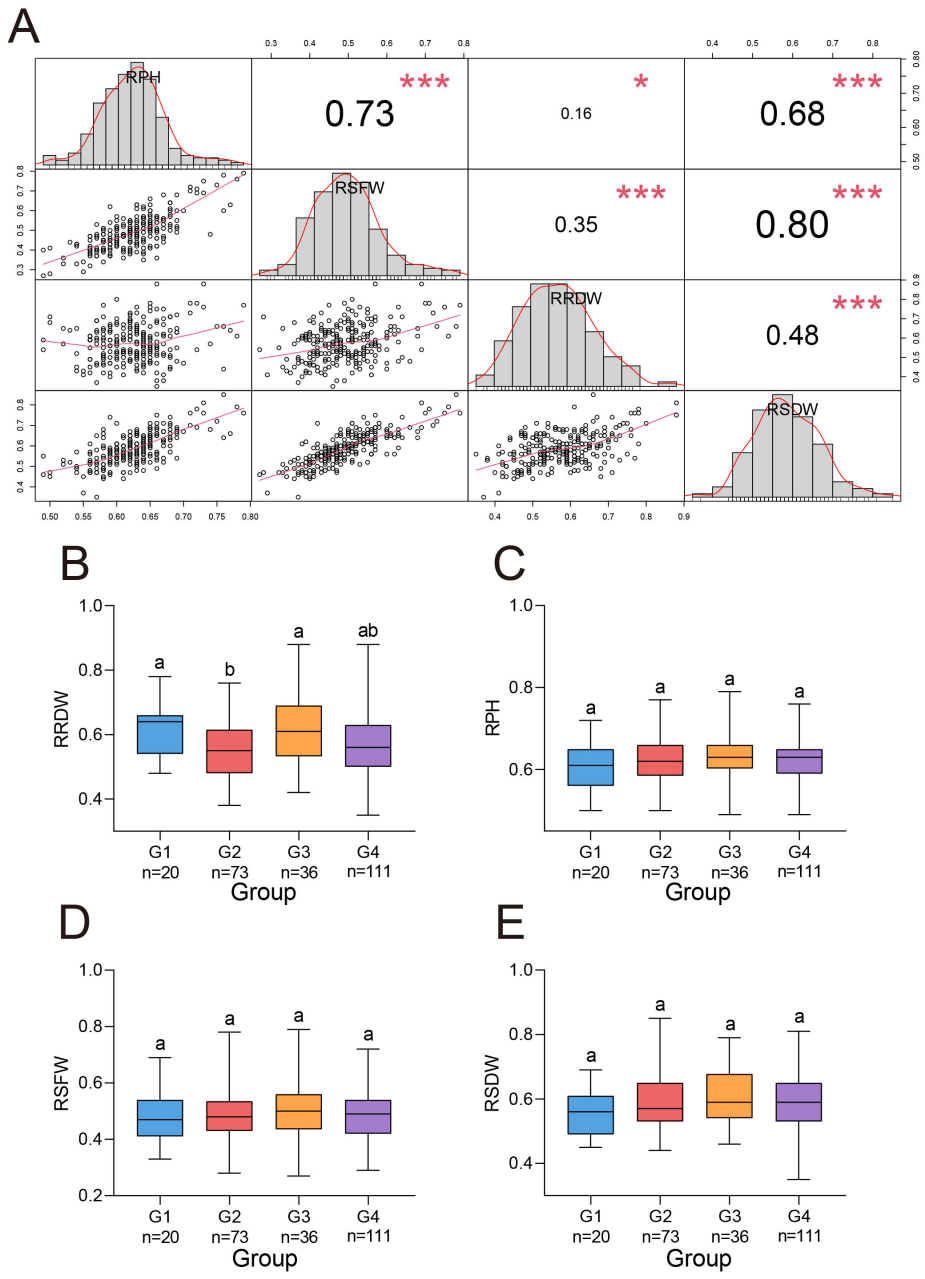
Four salt stress traits in *G. barbadense*. **(A)** Phenotypic correlation matrix (upper triangle) with diagonal histograms showing trait frequency distributions for 239 *G. barbadense* accessions. Correlation coefficients represent Pearson’s r values. Histograms display trait variance with normality distribution curves. * and *** indicated P value at the 0.05 and 0.001 levels, respectively. **(B-E)** Comparative analysis of normalized growth parameters across genetic subgroups (G1-G4): **(B)** RRDW, **(C)** RPH, **(D)** RSFW, **(E)** RSDW. n values: number of accessions within each subgroup. One-way analysis of variance (ANOVA) was performed to assess differences between subpopulations, significantly different (P < 0.05) groups are denoted by distinct lowercase letters.

All four traits exhibited a normal distribution across replicates under salt stress, indicating their suitability for GWAS analysis. These traits showed significant positive correlations between each other (**Figure 2A**). Among them, the strongest correlation was observed between RSFW and RSDW (r = 0.80), while the weakest correlation was found between RPH and RRDW (r = 0.16). In terms of RRDW, G3 showed significantly higher values than G2, and G1 performed significantly better than G2 (**Figure 2B**). For the other three traits, no significant differences were observed among the subgroups (**Figures 2C-E**). Therefore, under salt stress, the materials in subgroup G3 demonstrated better salt tolerance.

PCA was conducted on the four relative salt-tolerance indices of the 239 *G. barbadense* accessions. The eigenvalues and contribution rates of each principal component are shown in **Table 2**. Variance partitioning showed PC1 accounted for 66.98% (λ = 2.68), PC2 21.96% (λ = 0.88), PC3 6.53% (λ = 0.26), and PC4 4.53% (λ=0.18) of total variance. The cumulative contribution rate of the first two principal components reached 88.94%, which exceeds the threshold of 85% for selecting principal components. Therefore, the first two principal components can adequately represent the information from the original four traits. PC1 (66.98% variance) showed strongest positive loading for RSDW (loading = 0.35), followed by RSFW (0.34) and RPH (0.31). PC2 (21.96% variance) was predominantly loaded by RRDW (loading = 0.94) with negative correlation to RPH (-0.47) and RSFW (-0.17).

**Table 2:** Eigenvalues, variance contributions, and loading matrices of four PCA.

Factor	Principal component			
	1	2	3	4
Eigenvalue	2.68	0.88	0.26	0.18
Contribution rate/%	66.98	21.96	6.53	4.53
Accumulative Contribution rate/%	66.98	88.94	95.47	100.00
Relative plant height	0.31	-0.47	1.45	-0.07
Relative shoot fresh weight	0.34	-0.17	-0.89	1.58
Relative root dry weight	0.20	0.94	0.60	0.34
Relative shoot dry weight	0.35	0.05	-0.76	-1.70

**Figure 3:**
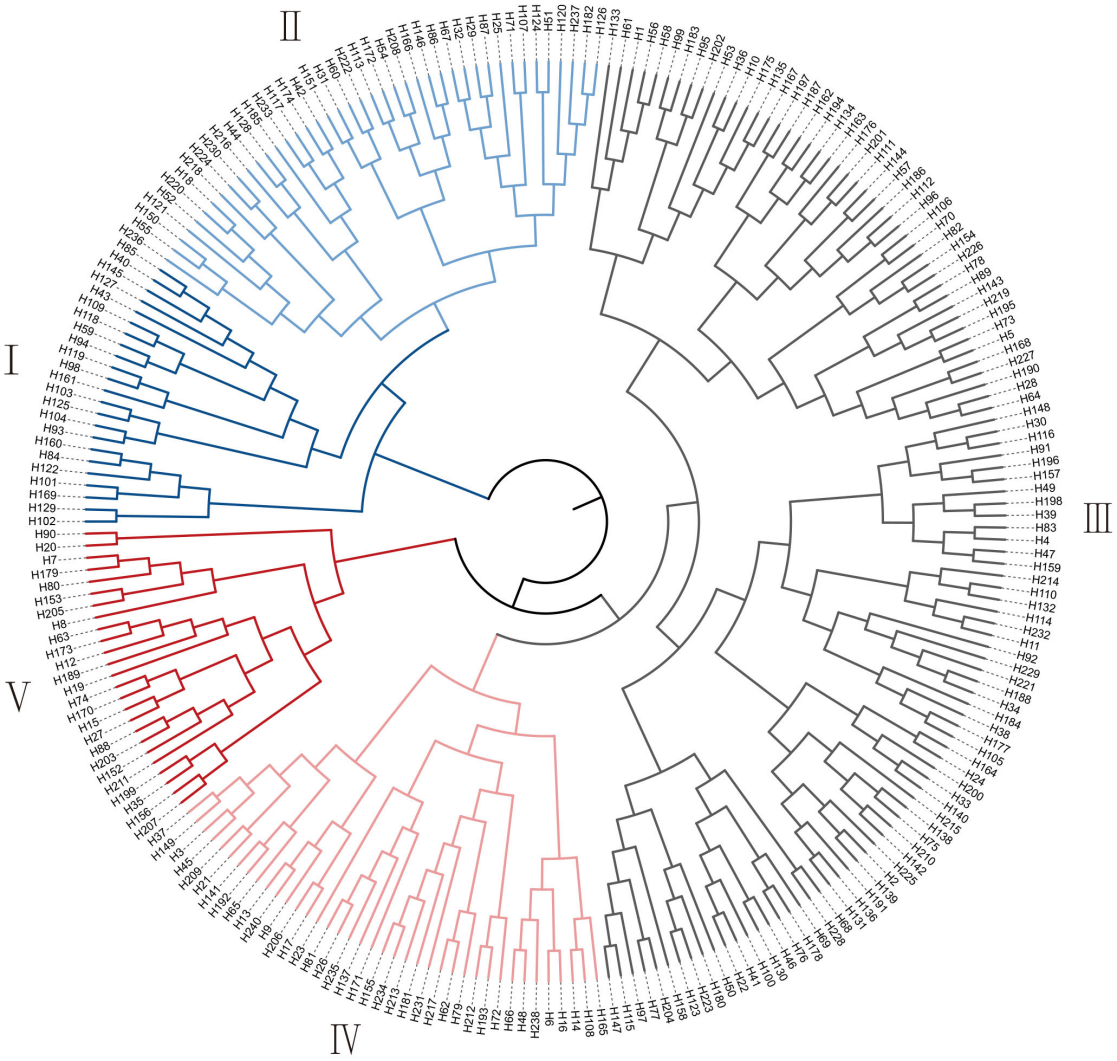
Clustering diagram for salt tolerance of 239 *G. barbadense* accessions.

Based on the contribution rates of the PC1 and PC2, the comprehensive salt tolerance index (D value) for the 239 *G. barbadense* accessions was calculated using membership function analysis. Hierarchical clustering was then performed to classify the accessions into five distinct groups (**Figure 3**, **Supplementary Table S4**): 23 highly salt-tolerant, 42 moderately salt-tolerant, 110 intermediate, 39 moderately salt-sensitive, and 25 highly salt-sensitive accessions, labeled as Groups I to V, respectively. The corresponding D value ranges for the five groups: I (0.63–0.91), II (0.52–0.61), III (0.38–0.51), IV (0.28-0.37), V (0.09-0.28). Group III (intermediate tolerance) comprised 46.0% of the panel (110/239), whereas Group I (high tolerance) represented only 9.6% (23/239), reflecting the polygenic nature of salt tolerance. This classification method facilitates the screening and identification of salt-tolerant *G. barbadense* accessions (Group I), providing a foundation for developing salt-tolerant *G. barbadense* cultivars.

### 3.4 Genome-wide association study of salt tolerance in *G. barbadense* population

Association analysis of four traits under two environments and breeding values was performed using the Mixed Linear Model (MLM). Based on the significance thresholds of *p* < 4.67×10^-6^ [1/n, where n = 213,990 effective SNPs calculated using PLINK software (Yu et al., 2021)] for SNPs detected in a single environment and *p* < 1×10^–5^ for repeatedly identifing SNPs, a total of 1,577 SNP loci were identified (**Supplementary Table S5**, **Supplementary Figure S5**). Among these, 34 SNPs were uniquely detected in single environments, while 1,543 SNPs were repeatedly identified across two or more environments or traits. Trait-specific associations showed varying genetic architectures: RPH (1,453 SNPs), RSFW (1,407), RSDW (41), RRDW (50). SNP distribution showed significant subgenome bias, with 90.8% (1,433/1,577) localized to the At subgenome versus 9.2% (144/1,577) in Dt. This disparity may be attributed to the higher linkage disequilibrium (LD) decay rate and larger LD blocks observed in the At subgenome (**Supplementary Figure S2**). LD decay intervals were employed to refine candidate gene selection. By defining 100-kb genomic regions upstream and downstream of significant SNPs as LD blocks (with overlapping regions merged), we identified 132 salt stress-related QTLs (**Supplementary Table S6**) spanning approximately 47.78 Mb collectively. The At subgenome containing 81.2% (38.80/47.78 Mb) of QTL regions versus 20.9% (9.98 Mb) in Dt. These QTL regions, representing ~2.3% of the total genome length, encompassed 811 annotated genes (**Supplementary Table S7**).

### 3.5 Identification of candidate genes

**Figure 4:**
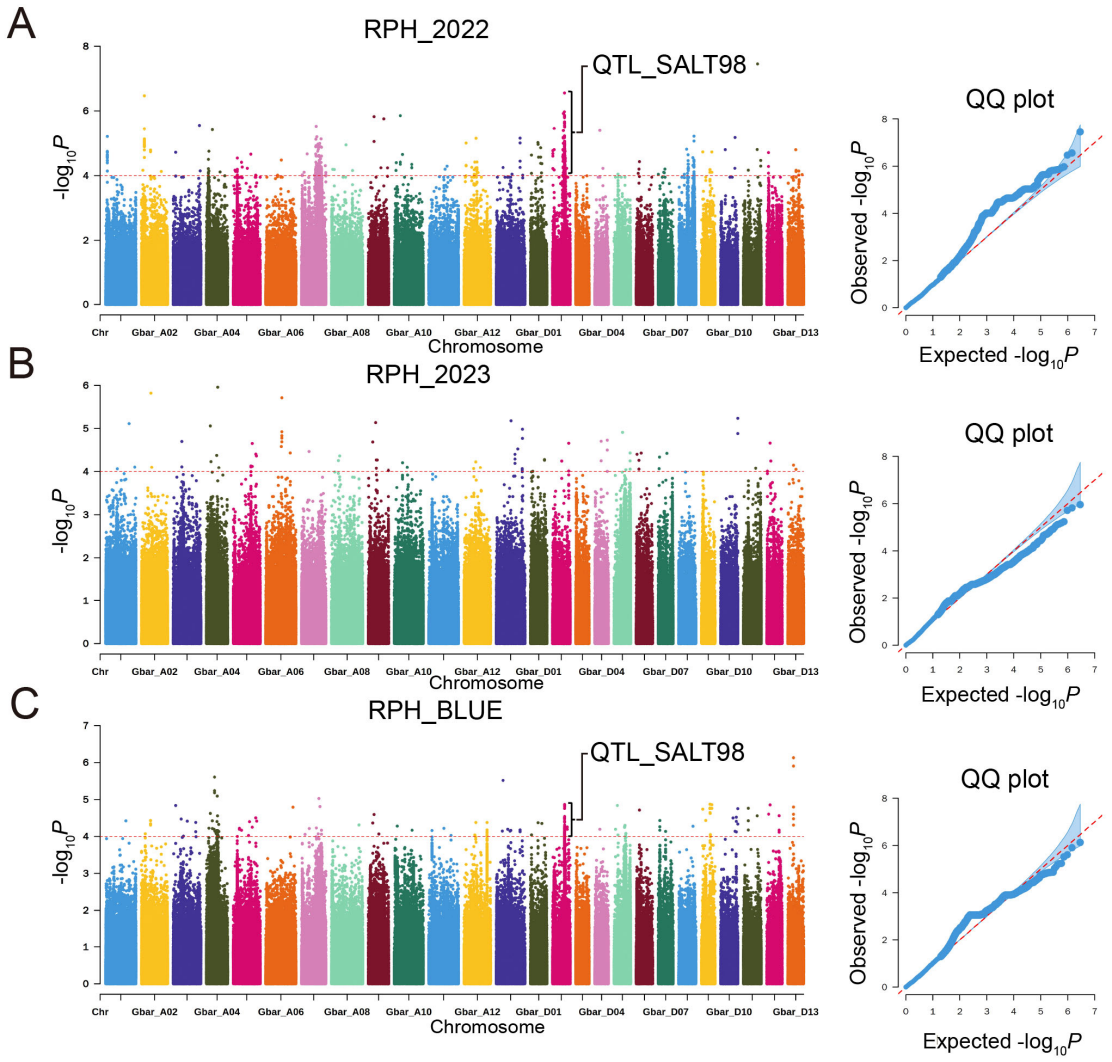
Manhattan and QQ plots for RPH in a GWAS of *G. barbadense*. **(A-C)** RPH: 2022 **(A)**, 2023 **(B)**, BLUE **(C)**.

**Figure 5:**
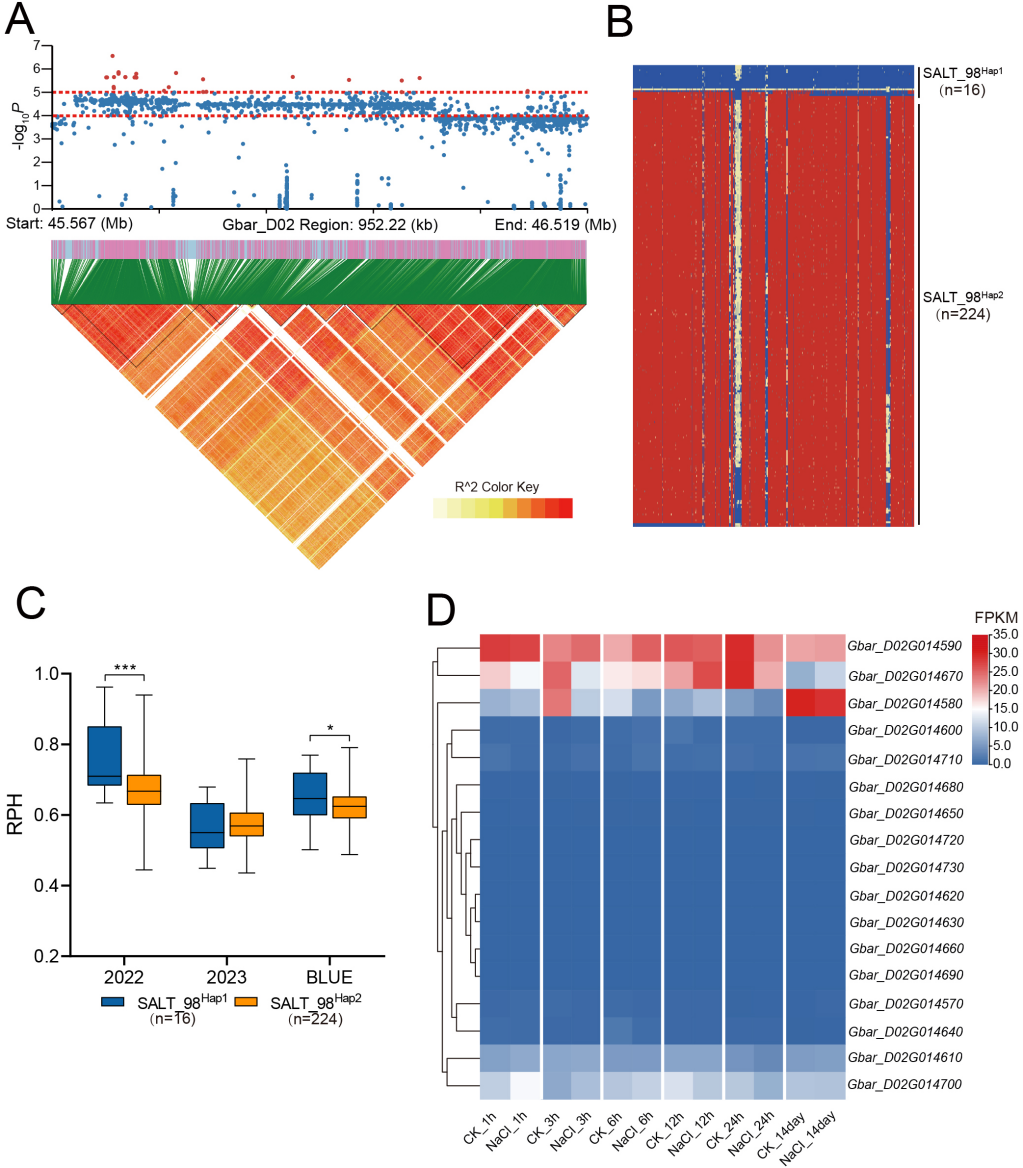
RPH-related loci were identified on D02. **(A)** Manhattan plot and LD block analysis. **(B)** Haplotype analysis within the CHR: D02: 45.56Mb-46.52Mb interval. **(C)** Box plots for RPH among different haplotypes. In the box plots, the center line denotes the median, box limits are the upper and lower quartiles, and whiskers mark the range of the data. Significance levels for inter-group differences: *P<0.05, ***P<0.001 (two-tailed Student’s t-test). **(D)** Heatmap of FPKM expression for genes within QTL-SALT98 under salt treatment at 1 h, 3 h, 6 h, 12 h, 24 h, and 14 day. n values, number of accessions in each haplotype.

GWAS of 2022-RPH and BLUE-RPH identified a significant SNP cluster on chromosome D02 (**Figure 4**), delineating the QTL-SALT98 locus (Gbar_D02: 45.56-46.52 Mb; 958 kb interval) spanning approximately 958 kb. The lead SNP (Gbar_D02_45674375) marked the association peak. Within QTL-SALT98, 1,596 SNPs were subjected to linkage disequilibrium (LD) analysis using LDBlockShow, which demonstrated strong linkage disequilibrium across this genomic region (**Figure 5A**). Haplotype analysis partitioned accessions into two major haplotypes (Hap1 and Hap2) (**Figure 5B**). Notably, Hap1 exhibited significantly higher RPH values compared to Hap2 (**Figure 5C**), and Hap1 also showed significantly higher RSFW, RSDW, and D value than Hap2 (**Supplementary Figure S6**), indicating a robust association between haplotype variation and salt tolerance capacity.

The QTL-SALT98 interval harbors 17 candidate genes. Analysis of published RNA-seq data from salt-stressed *G. barbadense* revealed dynamic expression patterns of these genes across 6 time points [1 h, 3 h, 6 h, 12 h, 24 h, and 14 days post-treatment (Hu et al., 2019; Dong et al., 2022)]. Notably, only five genes: *Gbar_D02G014580*, *Gbar_D02G014590*, *Gbar_D02G014610*, *Gbar_D02G014670*, and *Gbar_D02G014700* exhibited significant differential expression under salt stress (**Figure 5D**).

To validate candidate gene expression in extreme phenotypic materials, we selected the salt-tolerant genotype H160 and salt-sensitive genotype H20. Physiological characterization revealed contrasts in their salt tolerance. Under 7-day salt stress, H160 exhibited less structural alterations with mostly upright stems and partially turgid leaves, showing only slight wilting compared to controls, whereas H20 displayed severe wilting and pronounced stem bending (**Supplementary Figures S7A, B**). Growth parameters, including plant height, shoot biomass, and root dry weight, experienced moderate decreases in H160, in contrast to the significant declines observed in H20 under salinity (**Supplementary Figures S7C-F**). Ion profiling showed H160 roots retained 40% higher K^+^ content with a lower Na^+^/K^+^ ratio than H20, while its leaves maintained 40% lower Na^+^ accumulation and 45% reduced Na^+^/K^+^ ratio (**Supplementary Figures S7 G-I**), demonstrating coordinated regulation of Na^+^ exclusion in leaves and K^+^ retention in roots. These ion balance results indicate that H160 achieves salt tolerance by coordinating Na^+^ exclusion in leaves and K^+^ retention in roots, maintaining cellular ionic homeostasis critical for osmotic balance and enzyme function under salinity. In contrast, H20 failures to restrict Na^+^ accumulation in leaves and preserves root K^+^ levels, which leads to disrupted ion homeostasis and results in severe growth inhibition and wilted phenotype.

**Figure 6:**
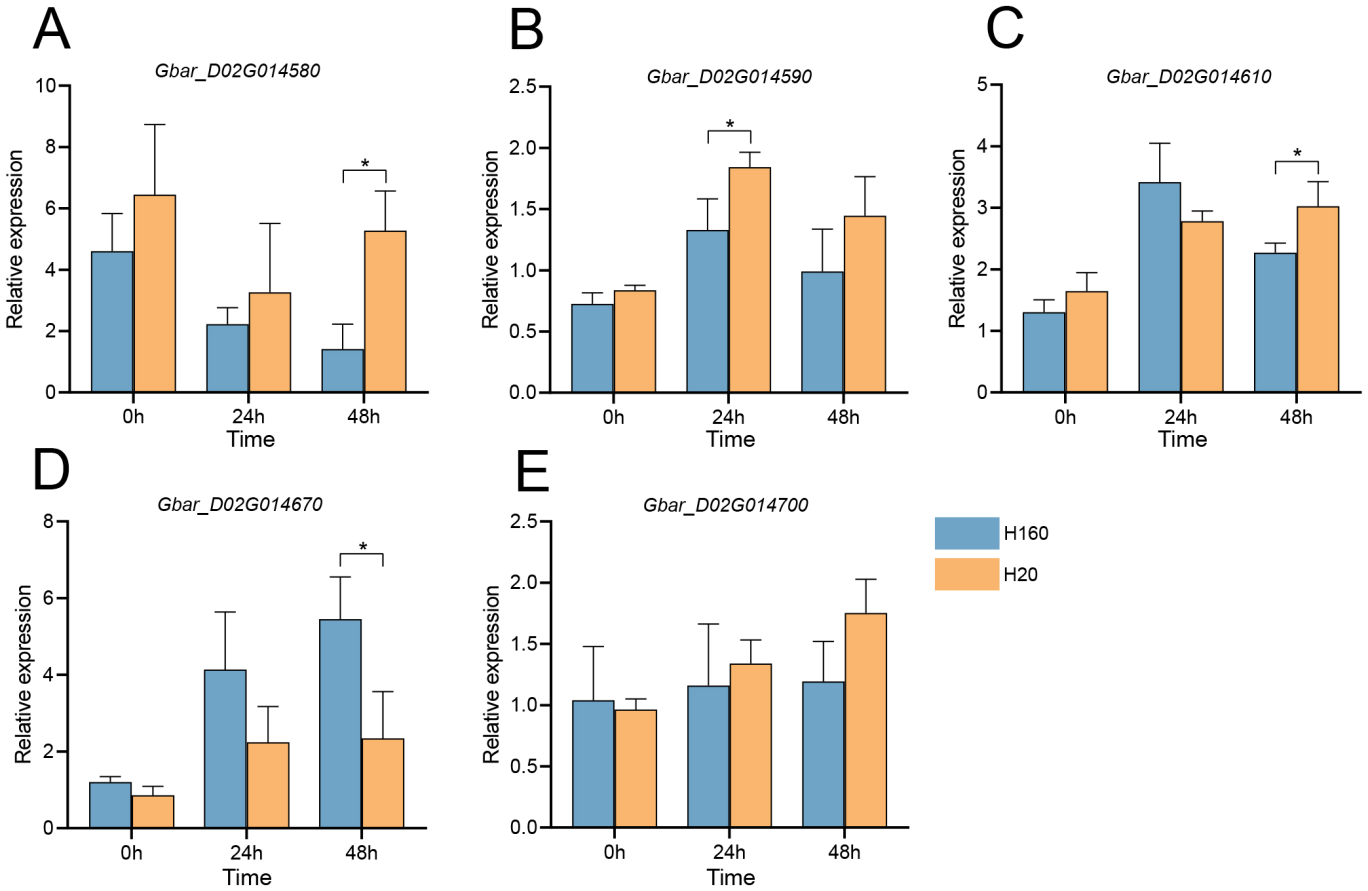
qRT-PCR analysis of five genes in the salt-tolerant material H160 and the salt-sensitive material H20 at 0 h, 24 h and 48 h under 200 mmol/L NaCl treatment. **(A)** *Gbar_D02G014580*. **(B)** *Gbar_D02G014590*. **(C)** *Gbar_D02G014610*. **(D)** *Gbar_D02G014670*. **(E)** *Gbar_D02G014700*. Expression levels were normalized to the housekeeping gene *UBQ7*. Significance levels for inter-group differences: *P<0.05 (two-tailed Student’s t-test).

Given these physiological disparities, we performed qRT-PCR validation at 0 h, 24 h, and 48 h post-treatment. Results demonstrated that *Gbar_D02G014580* and *Gbar_D02G014610* showed higher expression in H20 at 48 h, while *Gbar_D02G014590* exhibited elevated expression in H20 at 24 h (**Figures 6A-C**). Conversely, *Gbar_D02G014670* expression in H160 significantly surpassed that in H20 at both 24 h and 48 h, with the most pronounced difference observed at 48 h (**Figure 6D**). In addition, there was no significant difference in *Gbar_D02G014700* at each time (**Figure 6E**). These findings implicate *Gbar_D02G014590*, *Gbar_D02G014610*, *Gbar_D02G014670*, and *Gbar_D02G014700* as salt-responsive candidate genes. Functional annotation of Arabidopsis homologs revealed that *Gbar_D02G014670* (*AT2G01850*, *GbXTH27*) encodes a xyloglucan endotransglucosylase/hydrolase (XTH), which was critical for cell wall remodeling, while *Gbar_D02G014590* (*AT1G16020, GbCCZ1A*) encodes a vacuolar fusion protein, as a component of the MON1–CCZ1 complex, CCZ1A regulates post-Golgi vesicle transport to ensure targeted transport of storage proteins to protein storage vacuoles. *CCZ1A* dysfunction leads to seed development defects (Pan et al., 2021). *Gbar_D02G014610* (*AT1G24706*, *GbTHO2*) is a core component of the THO/TREX complex, which was essential for miRNA biogenesis. *Gbar_D02G014700* (*AT1G14710*) encodes a hydroxyproline-rich glycoprotein family protein. *Gbar_D02G014580* (*AT1G68020*, *GbTPS6*) encodes a trehalose-6-phosphatase catalyzing trehalose-6-phosphate (T6P) biosynthesis. T6P regulates sucrose biosynthesis, source-sink allocation, and developmental signaling in plants (Fichtner and Lunn, 2021).

### 3.6 Functional validation of *GbXTH27* in salt tolerance

**Figure 7:**
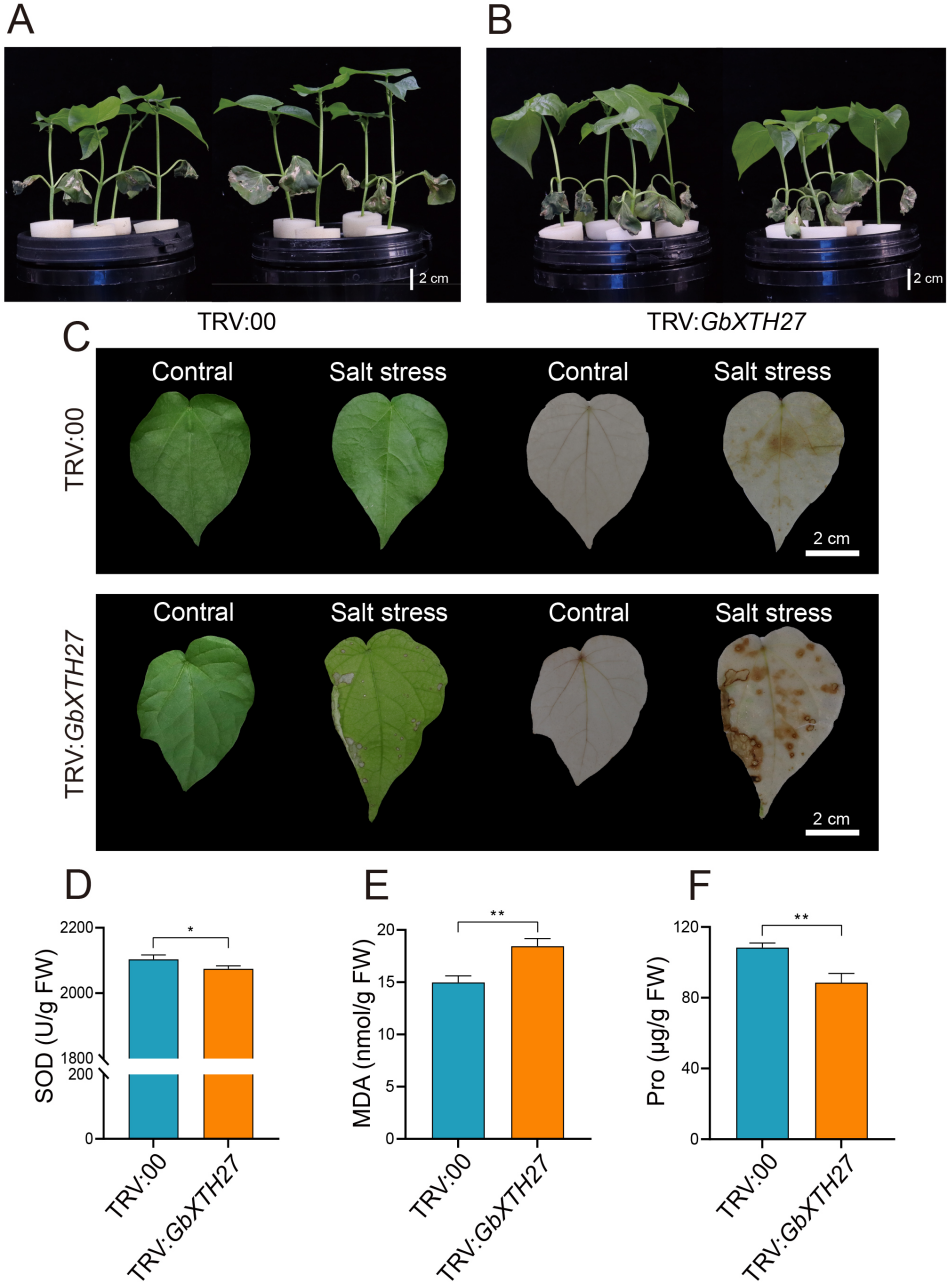
Gene silencing of *GbXTH27* in *G. barbadense*. Phenotypes under salt treatment, **(A)** TRV:00 control, **(B)** *GbXTH27* silenced. **(C)** DAB staining of TRV:00 and TRV: *GbXTH27* leaves with CK and salt stress. The green leaves are the images taken before the DAB staining. **(D)** SOD activity. **(E)** MDA content. **(F)** Pro content. Significance levels for inter-group differences: *P<0.05, **P<0.01 (two-tailed Student’s t-test). Bar = 2 cm.

VIGS of *GbXTH27* in the *G. barbadense* standard line 3–79 under salt stress (200 mM NaCl) were performed to verify its gene function. VIGS resulted in white-leaf phenotype (**Supplementary Figure S8A**) and significant transcript reduction (**Supplementary Figure S8B**). After 7 days of salt stress treatment, silenced plants (pTRV2: *GbXTH27*) displayed exacerbated wilting in cotyledons and true leaves compared to controls (TRV:00) (**Figures 7A, B**). Under salt stress conditions, TRV: *GbXTH27* showed significantly lower values in plant height, shoot fresh weight, shoot dry weight, and root dry weight than TRV:00 controls (**Supplementary Figures S9A-D**). Additionally, Na^+^ content in the roots, shoots, and leaves of TRV: *GbXTH27* was significantly higher than that in TRV:00 (**Supplementary Figure S9E**), while K^+^ content in the shoots and leaves of TRV: *GbXTH27* was significantly lower than in TRV:00 (**Supplementary Figure S9F**). Consequently, the Na^+^/K^+^ ratio in the roots, shoots, and leaves of TRV: *GbXTH27* was significantly higher than in TRV:00 (**Supplementary Figure S9G**), indicating that TRV: *GbXTH27* experienced more severe salt stress. After 7 days of NaCl treatment, the leaf DAB staining area of TRV: *GbXTH27* plants was significantly larger than that of TRV:00 controls, with deeper staining intensity in TRV: *GbXTH27*(**Figure 7C**). This indicates that silencing *GbXTH27* leads to a significant increase in reactive oxygen species (ROS) accumulation in leaves, significantly reducing the salt stress resistance of cotton seedlings. Physiological assays revealed diminished SOD activity and Pro content, alongside elevated MDA levels in silenced plants (**Figures 7D-F**), indicating compromised antioxidant capacity, membrane integrity, and osmotic adjustment. These results confirm *GbXTH27* as a key regulator of salt tolerance. The peak SNP (Gbar_D02_45674375) within QTL-SALT98 serves as a molecular marker for breeding salt-tolerant cotton cultivars.

## 4 Discussion

In this study, we performed a genetic structure analysis on 240 *G. barbadense* accessions, revealing the genetic diversity within the population and its complex geographic background. High-density molecular marker-based population structure analysis classified the population into four distinct subpopulations. Consistent with previous studies (Yu et al., 2021; Jin et al., 2023), Xinjiang *G. barbadense* accessions formed a separate cluster in the phylogenetic tree, exhibiting significant divergence from accessions of other regions. Notably, subpopulations G1 and G4 displayed marked differences in genetic diversity (**Figure 1D**). The higher genetic diversity of G1 may stem from its broader geographic distribution and limited artificial selection, whereas the reduced diversity in G4 likely reflects the restricted number of founder parents during the introduction of Xinjiang cultivars. Historical records indicate that Xinjiang *G. barbadense* varieties primarily originated from five Central Asian founder parents: 2И3, C6022, 8763И, 5230Ф, and 9122И (Zhao et al., 2022).

Salt stress significantly impairs growth-related traits, including reduced plant height, diminished leaf area, and suppressed root development, collectively leading to decreased biomass (Munns and Tester, 2008). Given the variability in salt tolerance mechanisms among accessions, relying on single or limited indicators may inadequately reflect the true salt-tolerance capacity (Zhang et al., 2011). To address this, we employed a multi-indicator approach for comprehensive evaluation. PCA of four stress tolerance indices (STIs) across 239 *G. barbadense* accessions enabled the calculation of a composite salt tolerance index (D-value) using membership functions. Higher D-values correlate with enhanced salt tolerance, providing a robust framework for comparative analysis. Clustering based on multiple agronomic traits offers superior discriminatory power over traditional methods in evaluating salt tolerance (Zeng et al., 2002). For instance, prior studies classified 549 *Brassica napus* accessions into five categories (highly tolerant, tolerant, intermediate, sensitive, and highly sensitive) using physiological traits (Wu et al., 2019), while eight wheat cultivars were grouped into salt-tolerant, moderately tolerant, and salt-sensitive categories (Quamruzzaman et al., 2022a). Adopting similar methodology, we classified 239 *G. barbadense* accessions into five groups, including highly tolerant, tolerant, intermediate, sensitive, and highly sensitive-based on D-values (**Figure 3**). The 23 highly tolerant accessions identified here represent valuable parental resources for salt-tolerant breeding.

GWAS have become crucial in dissecting salt tolerance in cotton. Current SNP identification strategies fall into two categories: (1) chip-based sequencing, for example, the detection of eight salt-associated SNPs in 288 *G. hirsutum* accessions by using an 80K chip (Cai et al., 2017), 23 SNPs linked to seedling traits in 713 *G. hirsutum* accessions via a 63K chip (Sun et al., 2018), and 42 SNPs identified in 149 *G. hirsutum* accessions using a 70K chip (Zheng et al., 2021); (2) whole-genome resequencing, which captures broader genetic variation. For example, resequencing of 419 *G. hirsutum* accessions uncovered 17,264 salt stress-associated SNPs, with key loci prioritized via linkage disequilibrium (LD) analysis (Yasir et al., 2019). Similarly, a MAGIC population comprising 550 recombinant inbred lines (RILs) enabled the identification of 23 salt tolerance-related QTLs across ~470,000 loci (Abdelraheem et al., 2021). While GWAS in cotton has predominantly focused on *G. hirsutum*, studies on *G. barbadense* remain limited. Here, resequencing of *G. barbadense* identified 2.98 million SNPs, constructing a high-density variation map. A total of 1,577 significant SNPs were detected, fewer than previous reports (Yasir et al., 2019; Xu et al., 2021), which may be due to stringent thresholds (*p* < 4.67 × 10^-6^ or *p* < 1.0 × 10^-5^ across two environments/traits) for minimizing the false positives.

GWAS analysis revealed multiple SNPs strongly associated with salt tolerance (**Supplementary Table S5**). Notably, no overlap was observed between the QTL intervals identified here and those reported in a prior GWAS of fiber phenotypes under salt stress in 249 *G. barbadense* accessions (Su et al., 2020). This divergence suggests distinct salt tolerance mechanisms between seedling and full-growth stages, as cotton is particularly vulnerable during germination, emergence, and early seedling development (Sharif et al., 2019). On chromosome D13, SNP Gbar_D13_54698281, associated with traits 2022-RPH and 2022-RSDW, which resides ~42 kb upstream of *Gbar_D13G020420*, an ortholog of Arabidopsis *AtCIPK6*. *GhCIPK6* regulates sugar homeostasis by interacting with *GhCBL2* and *GhTST2*, and its overexpression enhances salt tolerance in transgenic Arabidopsis (He et al., 2013; Deng et al., 2020). These findings highlight how our GWAS results can identify genes governing seedling-stage salt tolerance in *G. barbadense.*

The cell wall serves as the primary barrier against environmental stress, and its structural compromise can lead to membrane damage and ion homeostasis disruption (Zhu, 2016). To investigate the molecular basis of cell wall-mediated salt tolerance, this study identified *GbXTH27*, encoding a xyloglucan endotransglucosylase/hydrolase (XTH) with dual xyloglucan endotransglucosylase (XET) and xyloglucan endohydrolase (XEH) activities. XTHs mediate xyloglucan crosslinking, facilitating cell wall remodeling, which is a critical process for bridging primary and secondary cell walls. XTHs play conserved yet diverse roles in plant stress adaptation. Overexpression of *CaXTH3* from pepper (*Capsicum annuum*) in Arabidopsis and tomato enhances drought and salt tolerance by promoting stomatal closure via enhanced guard cell wall remodeling, thereby reducing transpirational water loss (Choi et al., 2011; Colin et al., 2023). Similarly, heterologous expression of *PeXTH* from *Populus euphratica* in tobacco increased palisade parenchyma cell density, reduced intercellular spaces, and enhanced leaf succulence, collectively lowering Na^+^ and Cl^-^ accumulation under salt stress (Han et al., 2013). These functional studies are supported by the characterization of XTH homologs in soybean (Song et al., 2018), wheat (Han et al., 2023), rapeseed (Chen et al., 2024), and maize (Fu et al., 2024). Similarly, heterologous expression of *PeXTH* from *Populus euphratica* in tobacco increased palisade parenchyma cell density, reduced intercellular spaces, and enhanced leaf succulence, collectively lowered Na^+^ and Cl^-^ accumulation under salt stress (Han et al., 2013). In this study, silencing *GbXTH27* resulted in severe wilting of cotton seedlings under salt stress (**Figures 7a, b**), ​​further supported by the observation that the leaf DAB staining area of TRV: *GbXTH27* plants was significantly larger than that of TRV:00 controls, with deeper staining intensity in TRV: *GbXTH27* (**Figure 7C**).​​ These phenotypic results collectively demonstrate that the loss of *XTH* function impairs salt tolerance. Furthermore, qRT-PCR analysis showed that under salt stress, the expression of *GbXTH27* in the salt-tolerant genotype H160 was significantly higher than that in the salt-sensitive genotype H20 (**Figure 6D**). This implies that *GbXTH27* may play a role in maintaining antioxidant enzyme activity and osmotic potential, facilitating cellular adaptation to salinity, possibly through mechanisms related to cell wall structural changes.

Physiological characterization revealed that *GbXTH27*-silenced plants displayed significantly reduced superoxide dismutase (SOD) activity (*p* < 0.05), indicative of impaired redox homeostasis, concurrent with elevated MDA accumulation (*p* < 0.01) characteristic of membrane lipid peroxidation (**Figures 7D, E**). Furthermore, Pro content was markedly reduced (*p* < 0.05), consistent with compromised osmotic adjustment capacity under salt stress. We propose two potential mechanisms underlying these observations: ROS accumulation via antioxidant suppression. Silencing *GbXTH27* likely suppress the activity of antioxidant enzymes such as superoxide dismutase (SOD) (p < 0.01; **Figure 7F**) (**Figure 7F**), leading to ROS accumulation and oxidative damage. On the other hand, osmotic adjustment was limited. Under salt stress, plants accumulate osmolytes like Pro to maintain turgor pressure (Szabados and Savoure, 2010). The significantly lower Pro content in *GbXTH27*-silenced lines (p < 0.01; **Figure 7F**) suggests that turgor-driven osmotic adjustment is restricted, thereby inhibiting Pro biosynthesis. These collective results demonstrate that *GbXTH27* critically mediates salt adaptation through regulation of redox homeostasis, reactive oxygen species (ROS) scavenging, and osmotic adjustment.

Through integrated analyses of population genetic structure, salt tolerance phenotyping, and GWAS in *G. barbadense* populations, we identified *GbXTH27* as a xyloglucan endotransglucosylase/hydrolase family gene whose expression positively correlates with salt tolerance levels. This study provides novel insights into the molecular mechanisms of salt stress adaptation in *G. barbadense* and highlights potential genetic targets for improving salt tolerance through molecular breeding.

## 5 Conclusion

This study integrates population genetic analysis, salt-tolerance phenotyping, and GWAS to elucidate the genetic diversity and salt adaptation mechanisms in *G. barbadense*. Additionally, 23 highly salt-tolerant accessions were identified through MFV. *GbXTH27* was identified, encoding an XTH enzyme, as a pivotal gene positively correlated with salt tolerance. Functional validation via VIGS confirmed its crucial role in enhancing seedling tolerance. These findings deepen our understanding of salt stress adaptation and offer genetic resources for cotton improvement of salt tolerant.

## Data Availability

The datasets presented in this study can be found in online repositories. The names of the repository/repositories and accession number(s) can be found in the article/**Supplementary Material**.

## Supplementary material

Supplementary Figure 1 The distribution of 2,983,855 SNPs and 369,812 InDels on the 26 chromosomes of the *G. barbadense* associated population.

Supplementary Figure 2 Decay of linkage disequilibrium with physical distance in the *G. barbadense* population. At sub-genome (black), complete accession set (red), and Dt sub-genome (blue).

Supplementary Figure 3 Heat map of kinship matrix of 240 *G. barbadense* accessions.

Supplementary Figure 4 CV error value among different K values.

Supplementary Figure 5 Manhattan and QQ plots for phenotypic traits in a GWAS of *G. barbadense*. **(A-C)** Relative shoot fresh weight (RSFW): 2022 **(A)**, 2023 **(B)**, BLUE **(C)**. **(D-F)** Relative shoot dry weight (RSDW): 2022 **(D)**, 2023 **(E)**, BLUE **(F)**. **(G-I)** Relative root dry weight (RRDW): 2022 **(G)**, 2023 **(H)**, BLUE **(I)**.

Supplementary Figure 6 Box plots for salt-relate traits among different haplotypes. **(A)** RSFW: relative shoot fresh weight. **(B)** RSDW: relative shoot dry weight. **(C)** RRDW: relative root dry weight. **(D)** D value. In the box plots, the center line denotes the median, box limits are the upper and lower quartiles, and whiskers mark the range of the data. Significance levels for inter-group differences: *P<0.05, **P<0.01 (two-tailed Student’s t-test).

Supplementary Figure 7 Growth pattern differences in phenotypes, biomass, and ion contents between salt-tolerant and salt-sensitive genotypes. **(A, B)** Phenotype of salt-tolerant H160 and salt-sensitive H20 under CK **(A)** and salt stress **(B)** for 7 days. **(C)** Plant height, **(D)** Shoot fresh weight, **(E)** Shoot dry weight, and **(F)** Root dry weight of H160 and H20 under CK and salt stress. **(G)** Na^+^ content, **(H)** K^+^ content and **(I)** Na^+^/K^+^ ratio in root, shoot, and leaf of H160 and H20. Significance levels for inter-group differences: *P<0.05, **P<0.01 (two-tailed Student’s t-test). Bar = 2 cm.

Supplementary Figure 8 VIGS silencing efficiency. **(A)** VIGS resulted in white leaf phenotype. **(B)** Relative expression of silenced *GbXTH27*. Significance levels for inter-group differences: *P<0.05 (two-tailed Student’s t-test).

Supplementary Figure 9 Phenotypes of TRV:00 and TRV: *GbXTH27* under control and salt stress treatment. **(A)** Plant height. **(B)** Shoot fresh weight. **(C)** Shoot dry weight. **(D)** Root dry weight. One-way analysis of variance (ANOVA) was performed to assess differences between subpopulations, significantly different (P < 0.05) groups are denoted by distinct lowercase letters. **(E)** Na^+^ content. **(F)** K^+^content. **(G)** Na^+^/K^+^ ratio. Significance levels for inter-group differences: *P<0.05, **P<0.01 (two-tailed Student’s t-test).
