# Supplementary figures and images for: Salt tolerance characterization and genome-wide association study of Gossypium barbadense accessions reveal salinity-adaptive variations
Source: Front Plant Sci. 2025 Sep 11;16:1654742. doi: 10.3389/fpls.2025.1654742 (PMC12460099; doi:10.3389/fpls.2025.1654742)

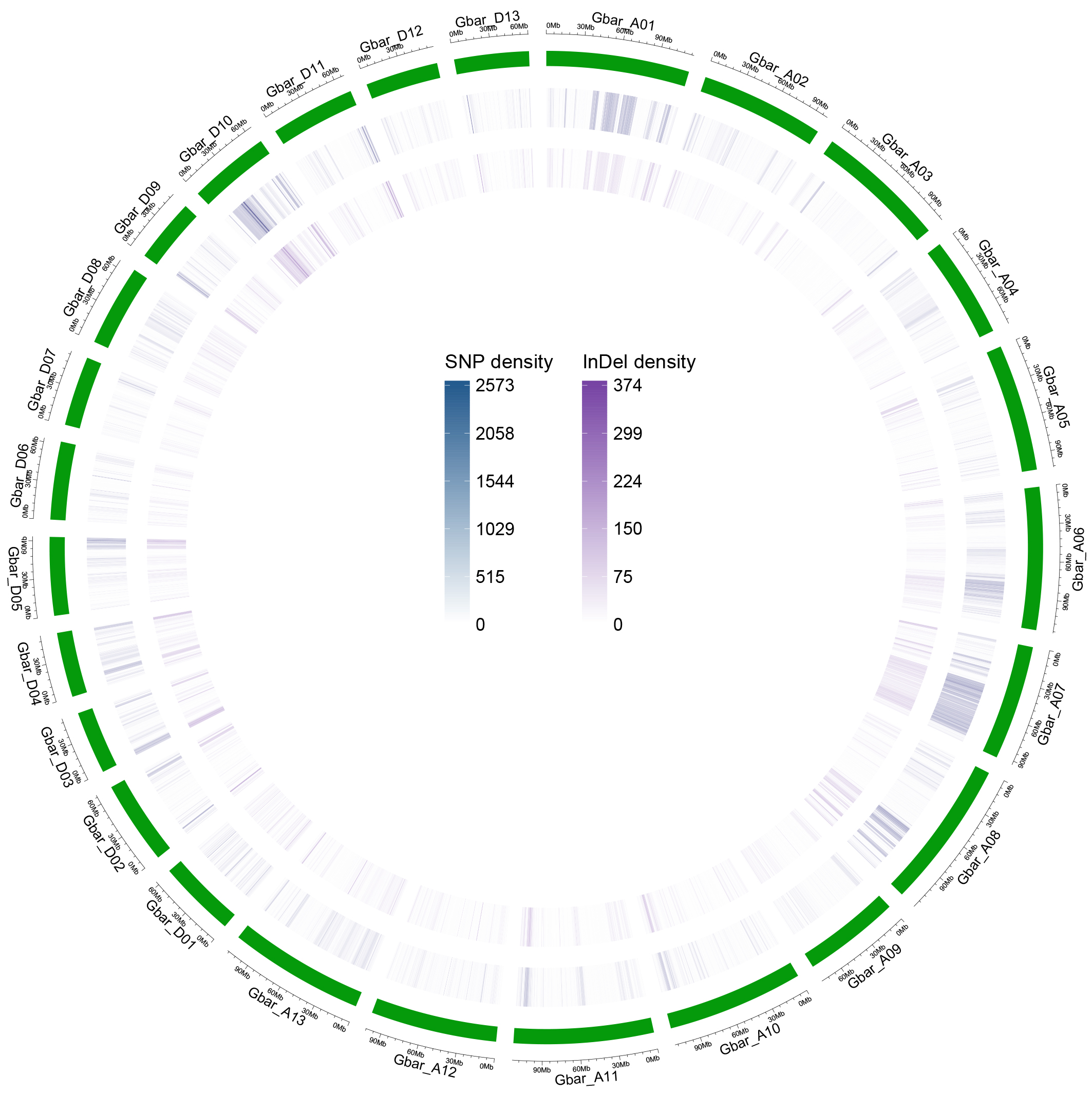

Supplement: Supplementary Figure 1 — The distribution of 2,983,855 SNPs and 369,812 InDels on the 26 chromosomes of the G. barbadense associated population. [file Image1.jpeg]

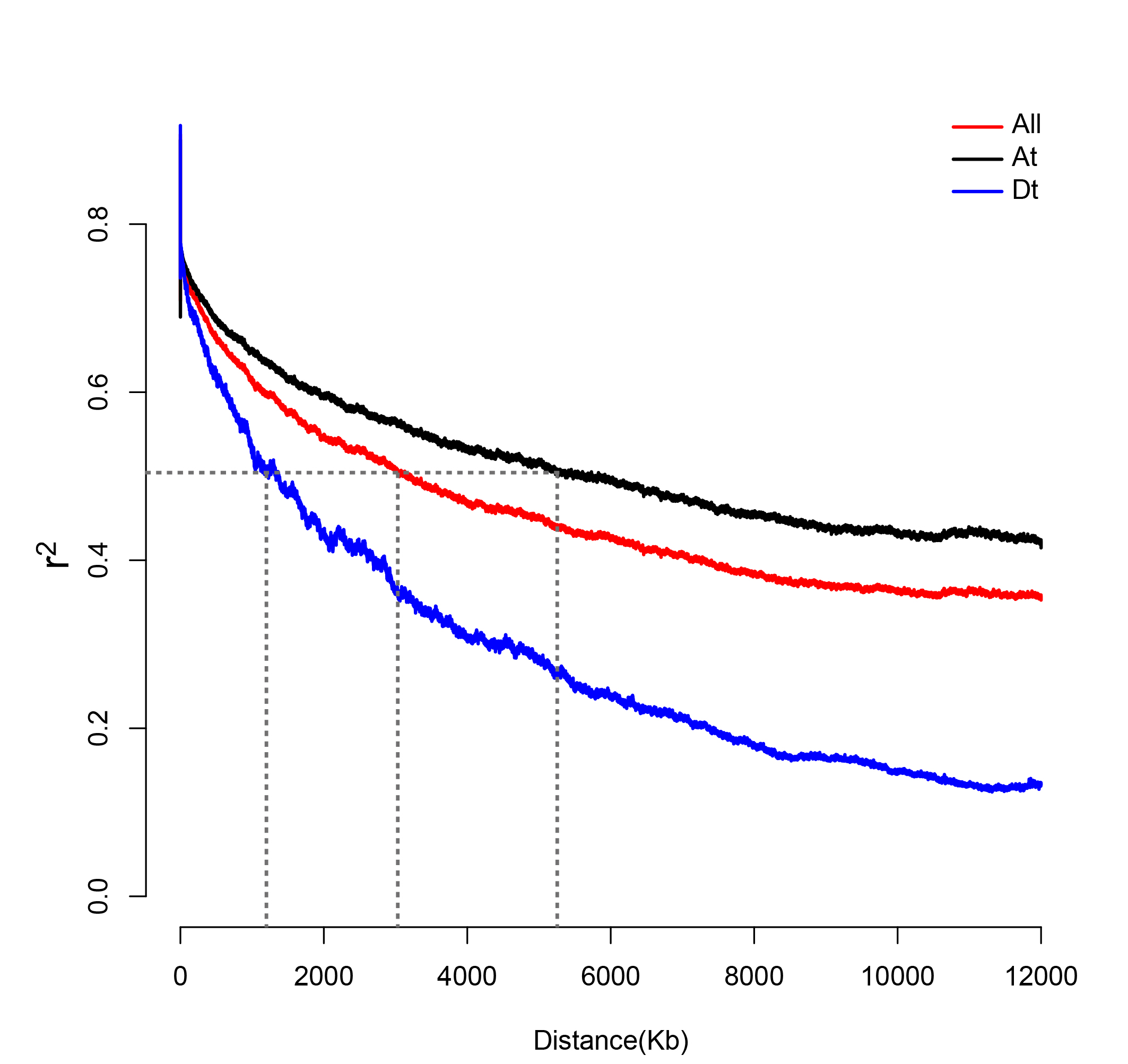

Supplement: Supplementary Figure 2 — Decay of linkage disequilibrium with physical distance in the G. barbadense population. At sub-genome (black), complete accession set (red), and Dt sub-genome (blue). [file Image2.jpeg]

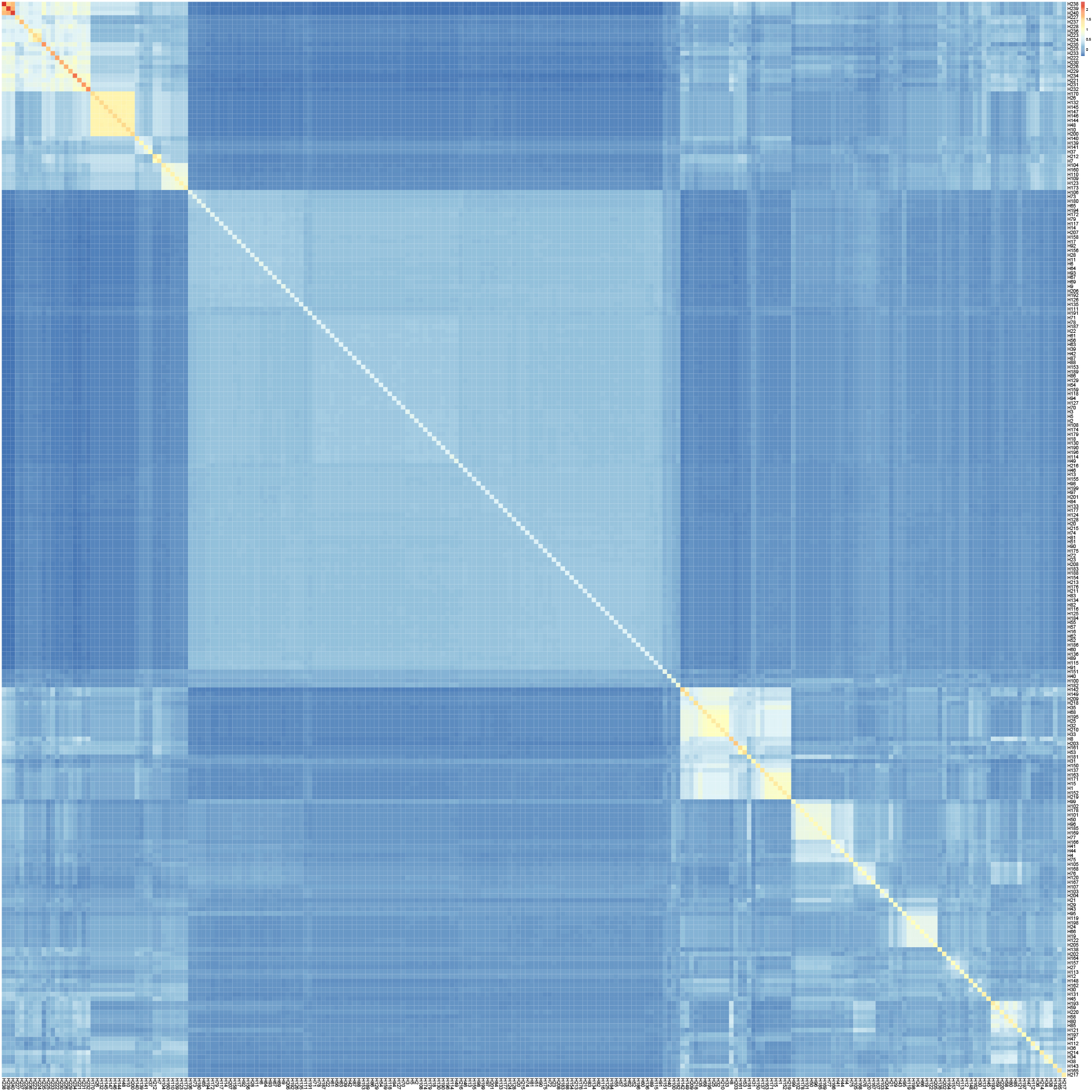

Supplement: Supplementary Figure 3 — Heat map of kinship matrix of 240 G. barbadense accessions. [file Image3.jpeg]

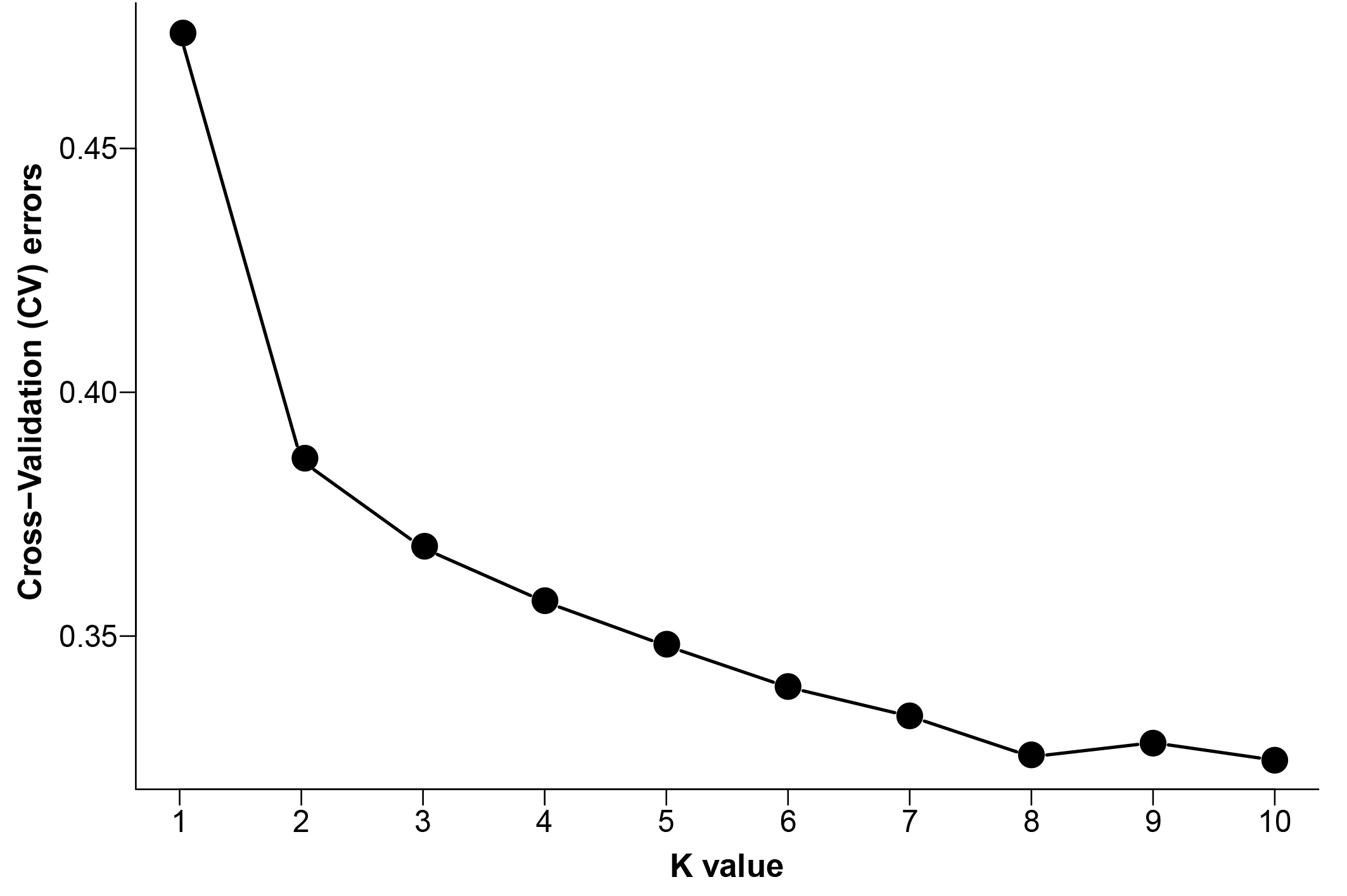

Supplement: Supplementary Figure 4 — CV error value among different K values. [file Image4.jpeg]

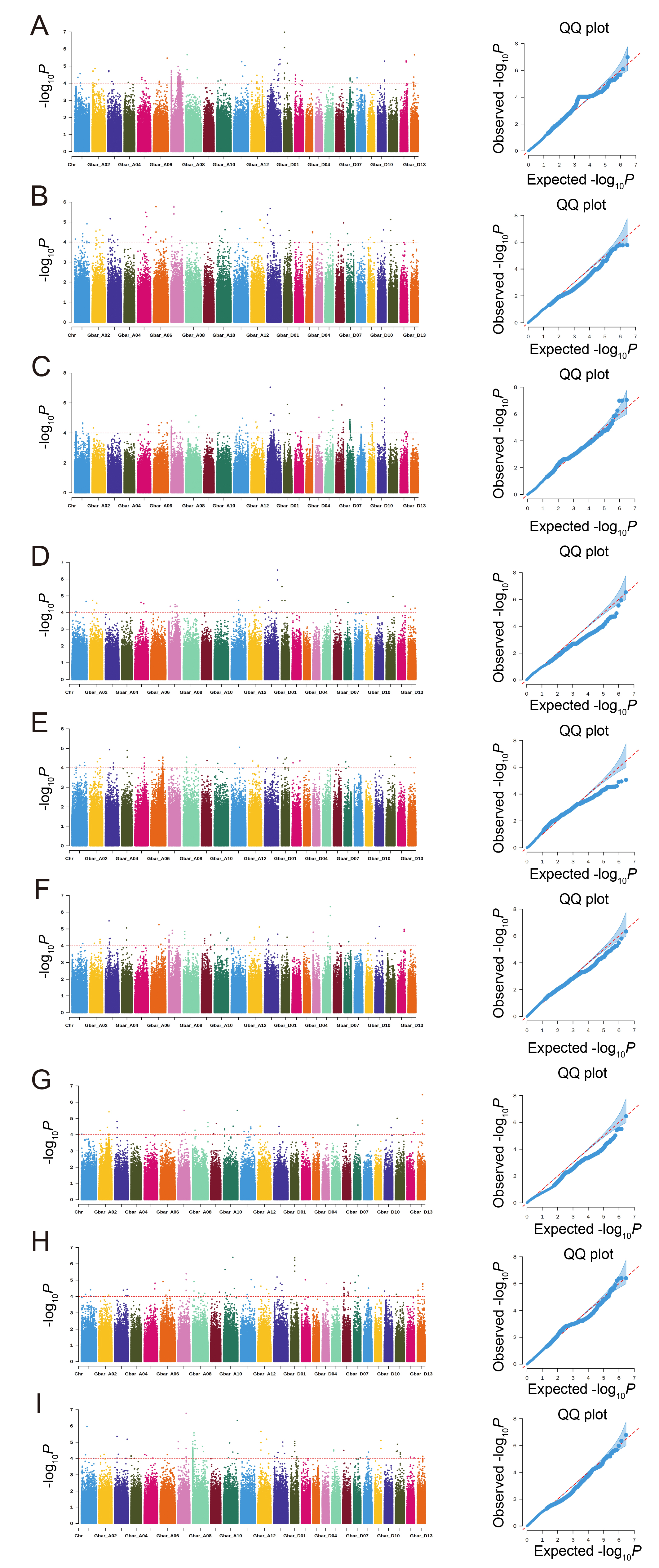

Supplement: Supplementary Figure 5 — Manhattan and QQ plots for phenotypic traits in a GWAS of G. barbadense. (A-C) Relative shoot fresh weight (RSFW): 2022 (A), 2023 (B), BLUE (C). (D-F) Relative shoot dry weight (RSDW): 2022 (D), 2023 (E), BLUE (F). (G-I) Relative root dry weight (RRDW): 2022 (G), 2023 (H), BLUE (I). [file Image5.jpeg]

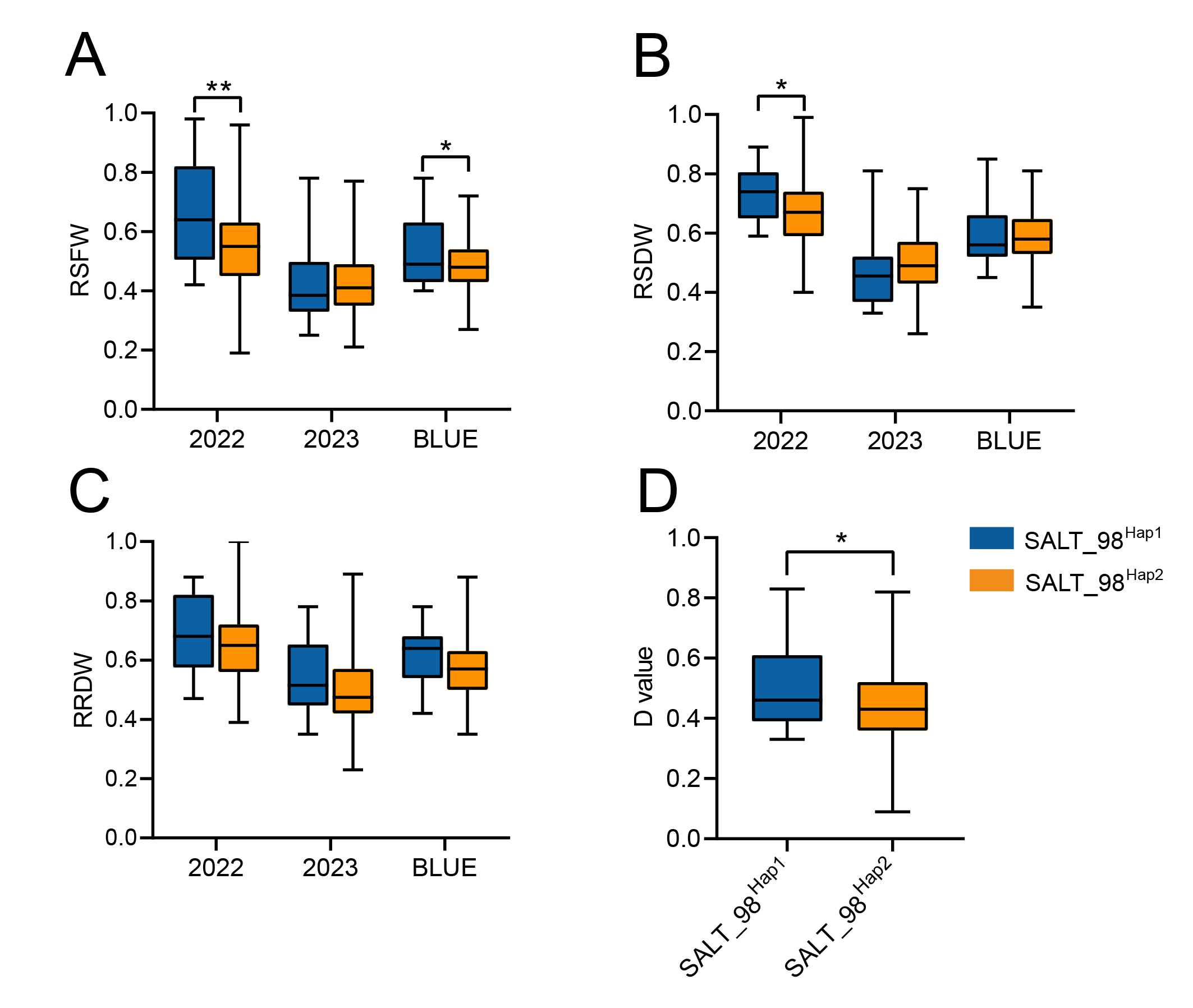

Supplement: Supplementary Figure 6 — Box plots for salt-relate traits among different haplotypes. (A) RSFW: relative shoot fresh weight. (B) RSDW: relative shoot dry weight. (C) RRDW: relative root dry weight. (D) D value. In the box plots, the center line denotes the median, box limits are the upper and lower quartiles, and whiskers mark the range of the data. Significance levels for inter-group differences: *P<0.05, **P<0.01 (two-tailed Student’s t-test). [file Image6.jpeg]

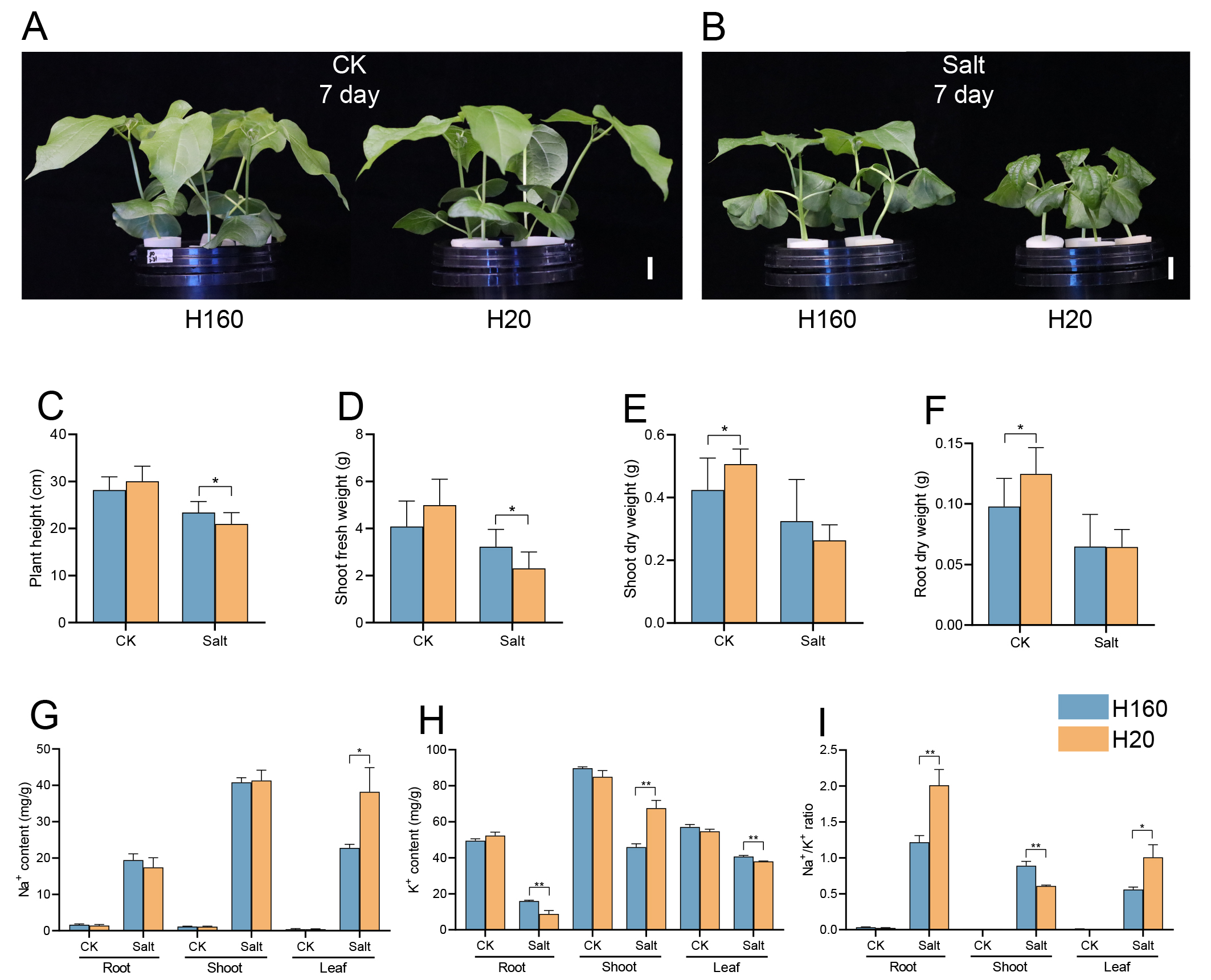

Supplement: Supplementary Figure 7 — Growth pattern differences in phenotypes, biomass, and ion contents between salt-tolerant and salt-sensitive genotypes. (A, B) Phenotype of salt-tolerant H160 and salt-sensitive H20 under CK (A) and salt stress (B) for 7 days. (C) Plant height, (D) Shoot fresh weight, (E) Shoot dry weight, and (F) Root dry weight of H160 and H20 under CK and salt stress. (G) Na+ content, (H) K+ content and (I) Na+/K+ ratio in root, shoot, and leaf of H160 and H20. Significance levels for inter-group differences: *P<0.05, **P<0.01 (two-tailed Student’s t-test). Bar = 2 cm. [file Image7.jpeg]

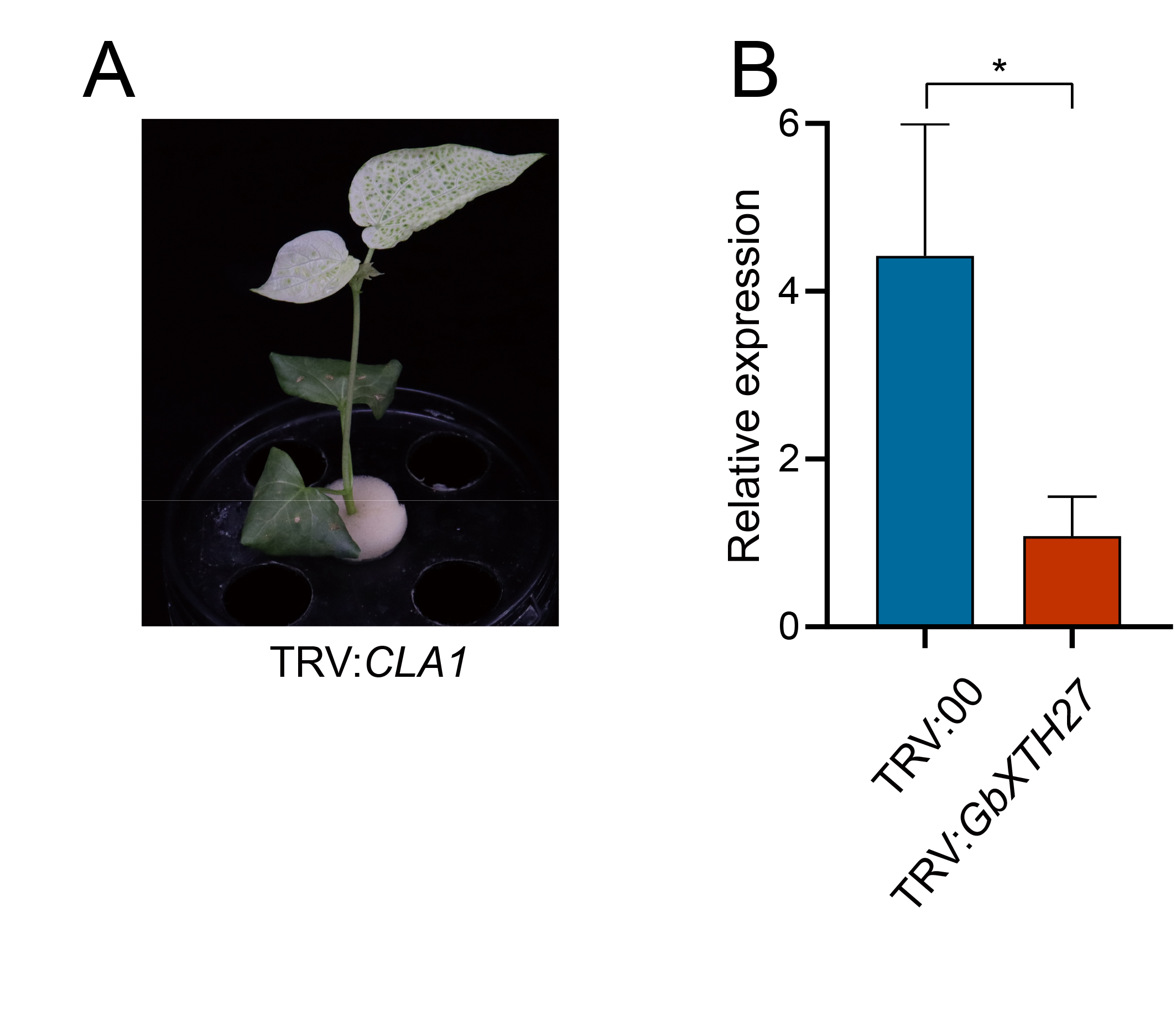

Supplement: Supplementary Figure 8 — VIGS silencing efficiency. (A) VIGS resulted in white leaf phenotype. (B) Relative expression of silenced GbXTH27. Significance levels for inter-group differences: *P<0.05 (two-tailed Student’s t-test). [file Image8.jpeg]

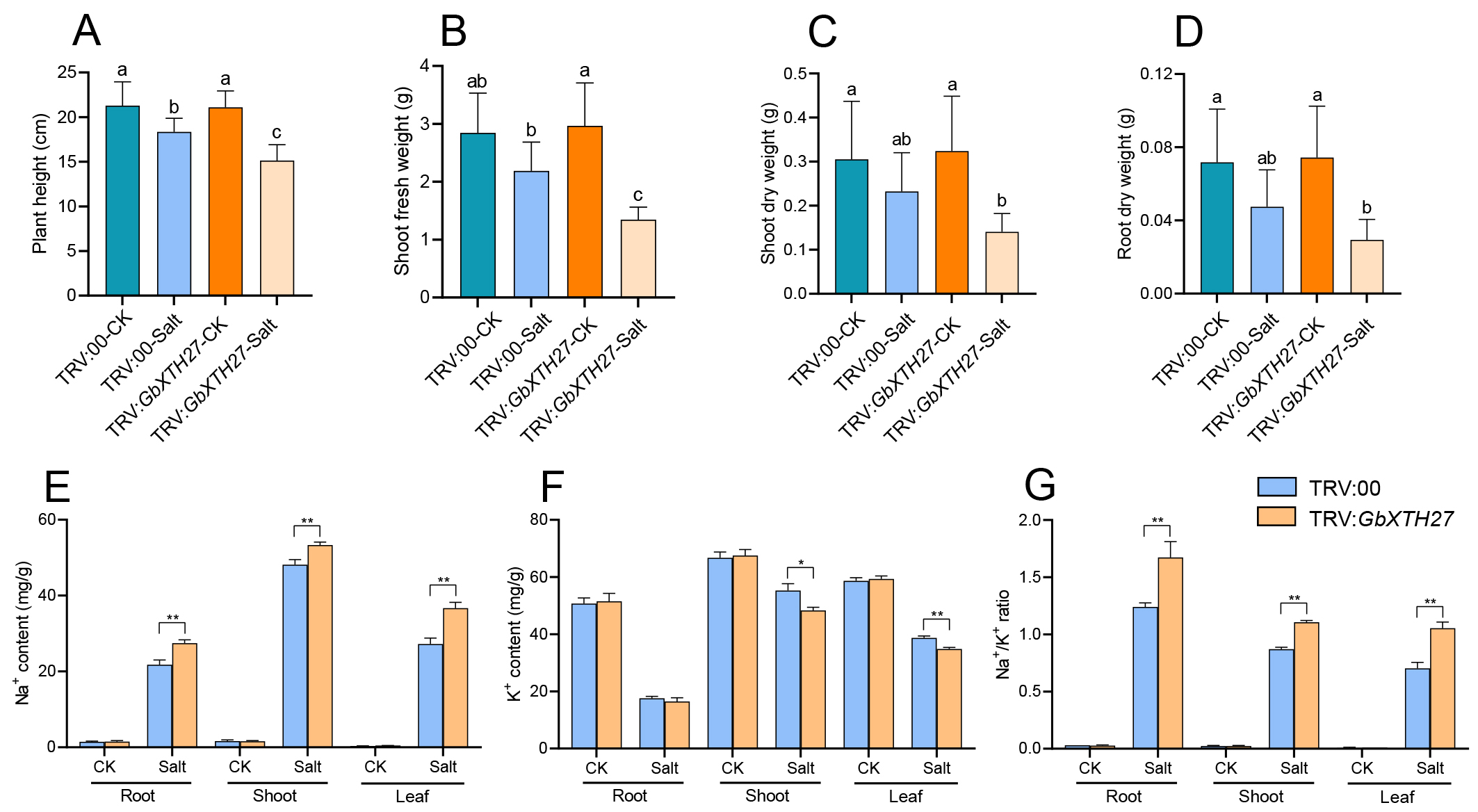

Supplement: Supplementary Figure 9 — Phenotypes of TRV:00 and TRV: GbXTH27 under control and salt stress treatment. (A) Plant height. (B) Shoot fresh weight. (C) Shoot dry weight. (D) Root dry weight. One-way analysis of variance (ANOVA) was performed to assess differences between subpopulations, significantly different (P < 0.05) groups are denoted by distinct lowercase letters. (E) Na+ content. (F) K+content. (G) Na+/K+ ratio. Significance levels for inter-group differences: *P<0.05, **P<0.01 (two-tailed Student’s t-test). [file Image9.jpg]
